# Triglycerides are an important fuel reserve for synapse function in the brain

**DOI:** 10.1038/s42255-025-01321-x

**Published:** 2025-07-01

**Authors:** Mukesh Kumar, Yumei Wu, Justin Knapp, Catherine L. Pontius, Daehun Park, Rose E. Witte, Rachel McAllister, Kallol Gupta, Kartik N. Rajagopalan, Pietro De Camilli, Timothy A. Ryan

**Affiliations:** 1 Department of Biochemistry, Weill Cornell Medicine, New York, NY USA; 2 Aligning Science Across Parkinson’s (ASAP) Collaborative Research Network, Chevy Chase, MD USA; 3 Department of Neuroscience, Howard Hughes Medical Institute, Program in Cellular Neuroscience, Neurodegeneration and Repair, Yale University School of Medicine, New Haven, CT USA; 4 Department of Cell Biology, Yale University School of Medicine, New Haven, CT USA; 5 Nanobiology Institute, Yale University, West Haven, CT USA; 6 Department of Internal Medicine, UT Southwestern Medical Center, Dallas, TX USA; 7 Laboratory of Molecular Genetics, Howard Hughes Medical Institute, Rockefeller University, New York, NY USA; 8 Department of Pharmacology, Yale University, New Haven, CT USA

**Keywords:** Cellular neuroscience, Lipids, Metabolism

## Abstract

Proper fuelling of the brain is critical to sustain cognitive function, but the role of fatty acid (FA) combustion in this process has been elusive. Here we show that acute block of a neuron-specific triglyceride lipase, DDHD2 (a genetic driver of complex hereditary spastic paraplegia), or of the mitochondrial lipid transporter CPT1 leads to rapid onset of torpor in adult male mice. These data indicate that in vivo neurons are probably constantly fluxing FAs derived from lipid droplets (LDs) through β-oxidation to support neuronal bioenergetics. We show that in dissociated neurons, electrical silencing or blocking of DDHD2 leads to accumulation of neuronal LDs, including at nerve terminals, and that FAs derived from axonal LDs enter mitochondria in an activity-dependent fashion to drive local mitochondrial ATP production. These data demonstrate that nerve terminals can make use of LDs during electrical activity to provide metabolic support and probably have a critical role in supporting neuron function in vivo.

## Abstract (web summary)

Kumar et al. show that under glucose-depleted conditions, neurons can use fatty acids as an alternative source of energy to support synaptic function.

## Main

Genetic studies of hereditary spastic paraplegias (HSPs) have identified over 80 different genetic loci associated with various forms of the disease^1–3^. Although the most common clinical presentation is associated with degradation of the cortico-spinal tract, a subset of patients with HSP present with a more complex phenotype associated with degradation in central nervous system function^4,5^. HSP54 is one such complex HSP, whereby patients present with a spectrum of central nervous system disorders, including intellectual disability, ataxias and vision impairment. The condition stems from mutations in DDHD2, which was subsequently shown to be a neuron-specific triglyceride (TG) lipase^6^. The loss of DDHD2 function results in the accumulation of LDs in mouse brain neurons^6^ and the appearance of a prominent lipid peak in cerebral proton magnetic resonance spectroscopy of human brains^7,8^.

In other metabolically demanding tissues, LDs serve as a TG storage depot. LD abundance reflects the balance of LD assembly and catabolic pathways; in the latter, liberated FAs enter mitochondria for β-oxidation^9^. Paradoxically, the brain, and in particular, neurons, have long been considered to rely almost solely on glucose for metabolic support without storage and dissipation of lipids as fuel; however, original experiments examining whether brain tissue can carry out fat-fuelled respiration^10–12^ used tissues that were not physiologically intact, and it was incorrectly assumed that acutely provided FAs would be as rapidly available for combustion as glucose. The dogma that neurons do not carry out β-oxidation was reinforced by the observation that LDs are rarely seen in healthy neurons. Furthermore, insight into neuronal FA flux and its metabolic outcome in terms of fuelling neuronal functions is limited because most previous studies focused on brain tissues that were a complex consortium of neurons and other cell types^11,13^. The discovery of DDHD2 as a neuron-specific TG lipase warrants a fresh examination of this long-held view, given that loss of its activity not only results in the buildup of LDs within neurons but also leads to cognitive impairment in both humans^8^ and mice^6^.

Here, we reveal a robust presence of DDHD2 lipase at synaptic terminals and show that blocking its activity leads to the accumulation of LDs at the majority of nerve terminals. Furthermore, using various metabolic and molecular sensors, we illustrate that electrical activity modulates DDHD2-mediated FA flux from LDs to mitochondria to drive ATP generation. We reveal that the metabolic support provided by FAs in turn can support synaptic vesicle (SV) recycling even in the absence of glucose. We show that in vivo, acute blockade of DDHD2 activity or the FA mitochondrial import machinery leads to a rapid onset of the torpor state in mice. Our study thus demonstrates that many neurons in the brain constantly rely on a steady flux of FAs derived from TGs to sustain function and provides mechanistic insights into DDHD2-mediated lipid metabolism in neurons.

## Results

### Neuron-specific DDHD2 lipase at hippocampal synapses

**Fig. 1:**
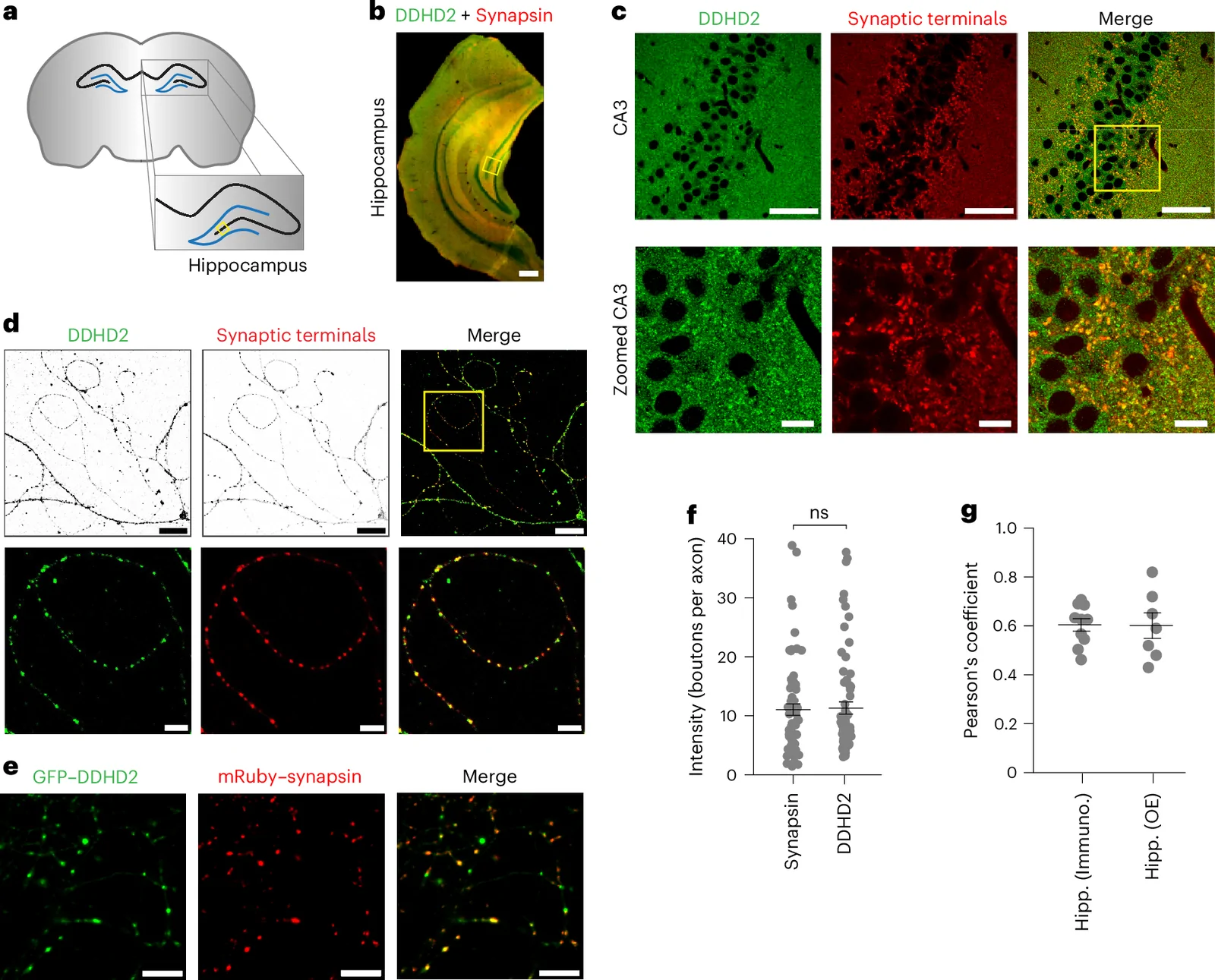
DDHD2 lipase localizes to synaptic terminals in the hippocampus. **a**, Schematic of a coronal section of rodent brain highlighting the hippocampus. The CA3 region is marked with a yellow box in the hippocampus. **b**, Laser scanned confocal micrograph of a brain hemisphere section near the mid-brain region, spanning the hippocampus, derived from a 28-day-old juvenile mouse brain, immunolabelled with DDHD2 and synapsin (synaptic terminal) antibodies. Scale bar, 400 µm. **c**, Confocal micrograph of the CA3 region (yellow box in **b**) of the hippocampus derived from a 28-day-old juvenile mouse brain, immunolabelled with synapsin (synaptic terminal) and DDHD2 antibodies. Scale bar, 50 µm. The lower panel shows the zoomed-in region (yellow box in upper right panel) of CA3. Scale bar, 10 µm. **d**, Confocal micrographs of dissociated hippocampal neurons immunolabelled with DDHD2 and synapsin antibodies. Scale bar, 20 µm. The lower panel shows the zoomed-in region (yellow box in upper right panel). Scale bar, 5 µm. **e**, Confocal micrographs of dissociated hippocampal neurons expressing GFP–DDHD2 and mRuby–synapsin. Scale bar, 10 μm. **f**, Quantification of relative DDHD2 and synapsin enrichment at synaptic terminals compared to adjacent axonal processes of hippocampal neurons in **d**. Data are presented as means; error bars, s.e.m. *P* value (ns, *P* = 0.501) was determined using an unpaired sample two-tailed *t*-test with *n* = 69 for DDHD2 and *n* = 64 for synapsin. **g**, Colocalization analysis of immunolabelled DDHD2 and synapsin (65 µm × 65 µm, *n* = 10), and GFP–DDHD2 and mRuby–synapsin (135 µm × 135 µm, *n* = 7) in hippocampal neurons, represented by Pearson’s correlation coefficient. Data are presented as means; error bars, s.e.m. Hipp., hippocampus; Immuno., immunostaining (of DDHD2 and synapsin); OE, overexpression (of GFP–DDHD2 and mRuby–synapsin). Source data

**Extended Data Fig. 1:**
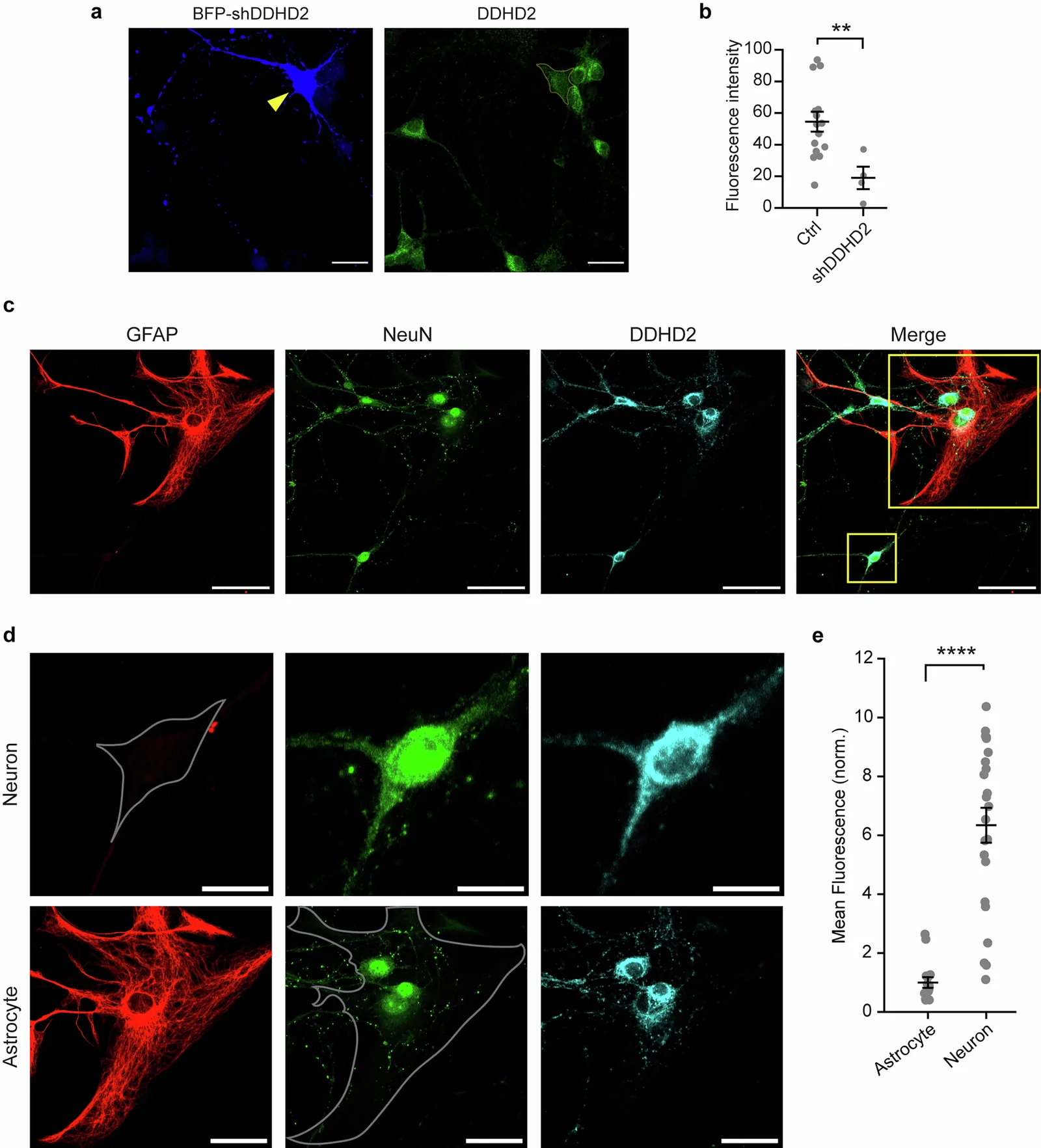
Validation of DDHD2 antibody specificity and localization in cell type. **a**, Confocal micrographs of BFP-shDDHD2 expressing hippocampal neurons immunolabelled with DDHD2 antibody. The representative shRNA expressing neuron (yellow arrowhead) shows reduced intensity of DDHD2 compared to non-expressing neurons in the same field of view. Scale bar: 20 μm. **b**, Quantification of DDHD2 fluorescence intensity in the soma of control (shRNA non-expressing) and transfected (DDHD2-specific shRNA expressing) neurons. Data are presented as mean ± SE. *p-value* (**p = 0.0092) was determined using unpaired samples two-tailed *t*-test with n = 14 for Ctrl and n = 4 for shDDHD2. **c**, Confocal micrographs of astrocytes (red, GFAP) and hippocampal neurons (green, NeuN) immunolabeled with DDHD2 (cyan) antibody. Scale bar: 50 μm. **d**, Zoomed-in regions of neuron and astrocyte marked with yellow boxes in (**c**). Scale bars, neuron: 10 μm, astrocyte: 30 μm. **e**, Quantification of fluorescence intensities of DDHD2 in neurons compared to astrocytes. Data are presented as mean ± SE. *p*-value (****p < 0.0001) was determined using unpaired samples two-tailed *t*-test with n = 15 for astrocytes and n = 23 for neurons. Source data

Synapses are crucial sites of motor coordination and memory formation. Depletion of DDHD2 lipase activity in neurons results in impaired motor and cognitive performance^6^, underscoring its functional importance at synapses. Consistent with the fact that genetic deletion of DDHD2 leads to massive accumulation of LDs in mouse neurons, immunocytochemistry with a DDHD2-specific antibody in acute brain slices (Fig. 1a) shows that this enzyme is present throughout the hippocampus (Fig. 1b). The staining was evident in the large mossy fibre synaptic terminals in CA3, as visualized by counter-immunostaining for synapsin, a presynaptic marker (Fig. 1c). To further characterize the subcellular localization of DDHD2 and determine its distribution in axons, we carried out immunocytochemistry analysis in sparsely plated dissociated primary rat hippocampal neurons, which showed that in axons, DDHD2 strongly co-localizes with synapsin in nerve terminals (Fig. 1d,f,g). Neurons in which DDHD2 was ablated (by small hairpin RNA (shRNA) expression) showed much lower levels of staining (Extended Data Fig. 1a,b), confirming the specificity of the antibody. Moreover, co-expression of GFP–DDHD2 and mRuby–synapsin showed strong colocalization in hippocampal neurons (Fig. 1e), with a Pearson’s coefficient of 0.60 ± 0.048 (Fig. 1g).

**Extended Data Fig. 2:**
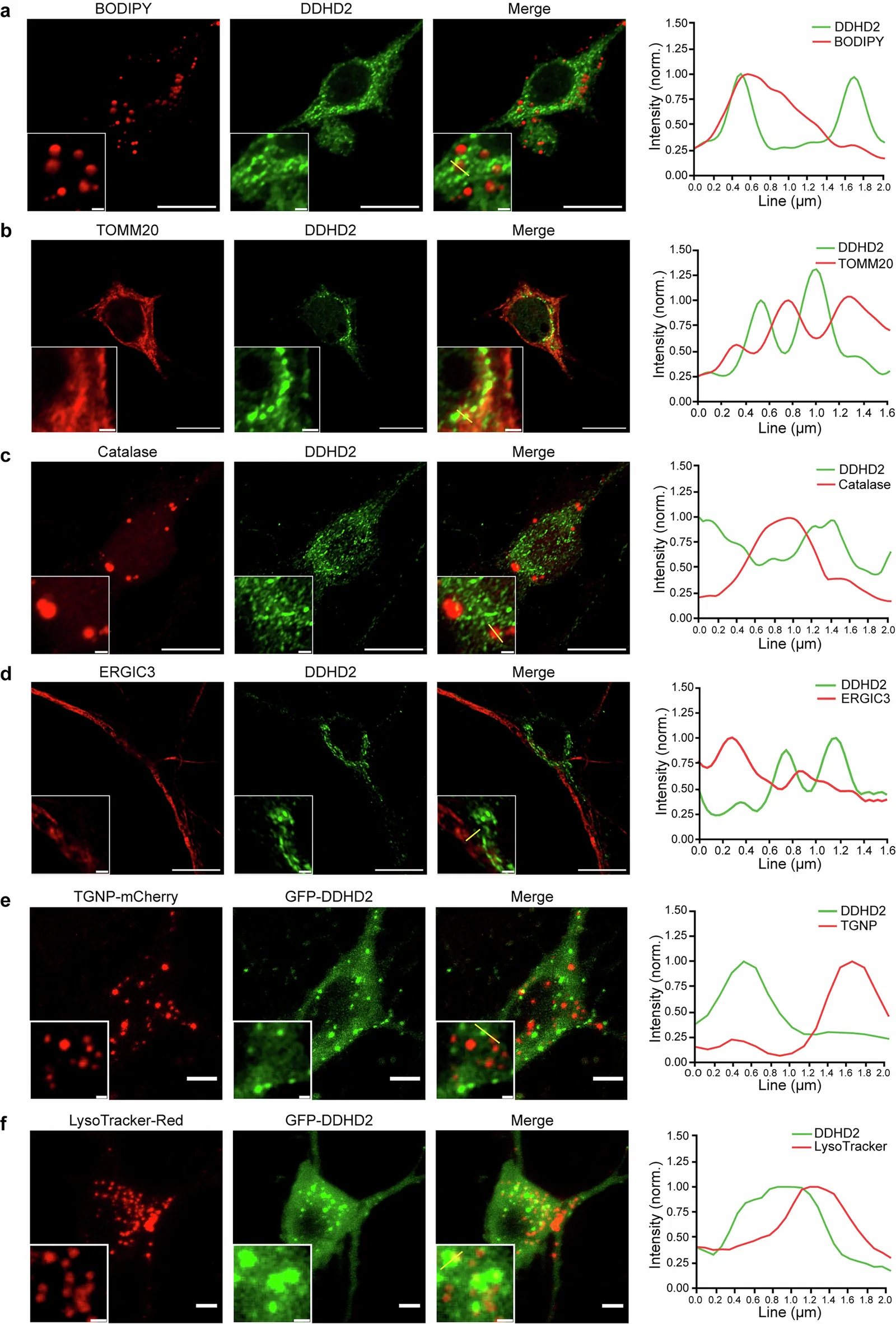
Subcellular localization of DDHD2. **a**, Confocal micrographs of hippocampal neurons treated with KLH45, immunostained for DDHD2, and stained with BODIPY to visualize lipid droplets. **b-d**, Confocal micrographs of hippocampal neurons co-immunostained for DDHD2 and organelle-specific markers: TOMM20 (mitochondria), catalase (peroxisomes), and ERGIC3 (ER-Glogi intermediate compartment). Scale bars: 10 μm; Zoomed-in panels: 1 μm. **e**, Confocal micrographs of hippocampal neurons expressing GFP-DDHD2 and TGNP-mCherry, showing their subcellular distribution. **f**, Confocal micrographs of hippocampal neurons expressing GFP-DDHD2 and stained with LysoTracker-Red to visualize lysosomes. Scale bars: 5 μm; Zoomed-in panels: 1 μm. Line-profile intensity graphs shown on the right correspond to the yellow lines drawn in the zoomed-in panels. Source data

Using a knockdown-validated DDHD2 antibody, we showed that the DDHD2 enzyme is over sixfold more enriched in neurons than in astrocytes (Extended Data Fig. 1c–e). In agreement with a previous report^14^, a fraction of DDHD2 puncta were found to be associated with LDs in KLH45-induced hippocampal neurons (Extended Data Fig. 2a). Interestingly, immunostaining revealed that DDHD2-containing structures are also closely appressed to mitochondria and lysosomes (Extended Data Fig. 2b,f), suggesting a functional correlation with the organelles. Therefore, we conclude that DDHD2 is broadly expressed and distributed throughout neurons, with particularly high enrichment in the nerve terminals within axons, supporting its potential role in synaptic lipid metabolism.

### Inhibition of DDHD2 causes accumulation of LDs at synapses

**Fig. 2:**
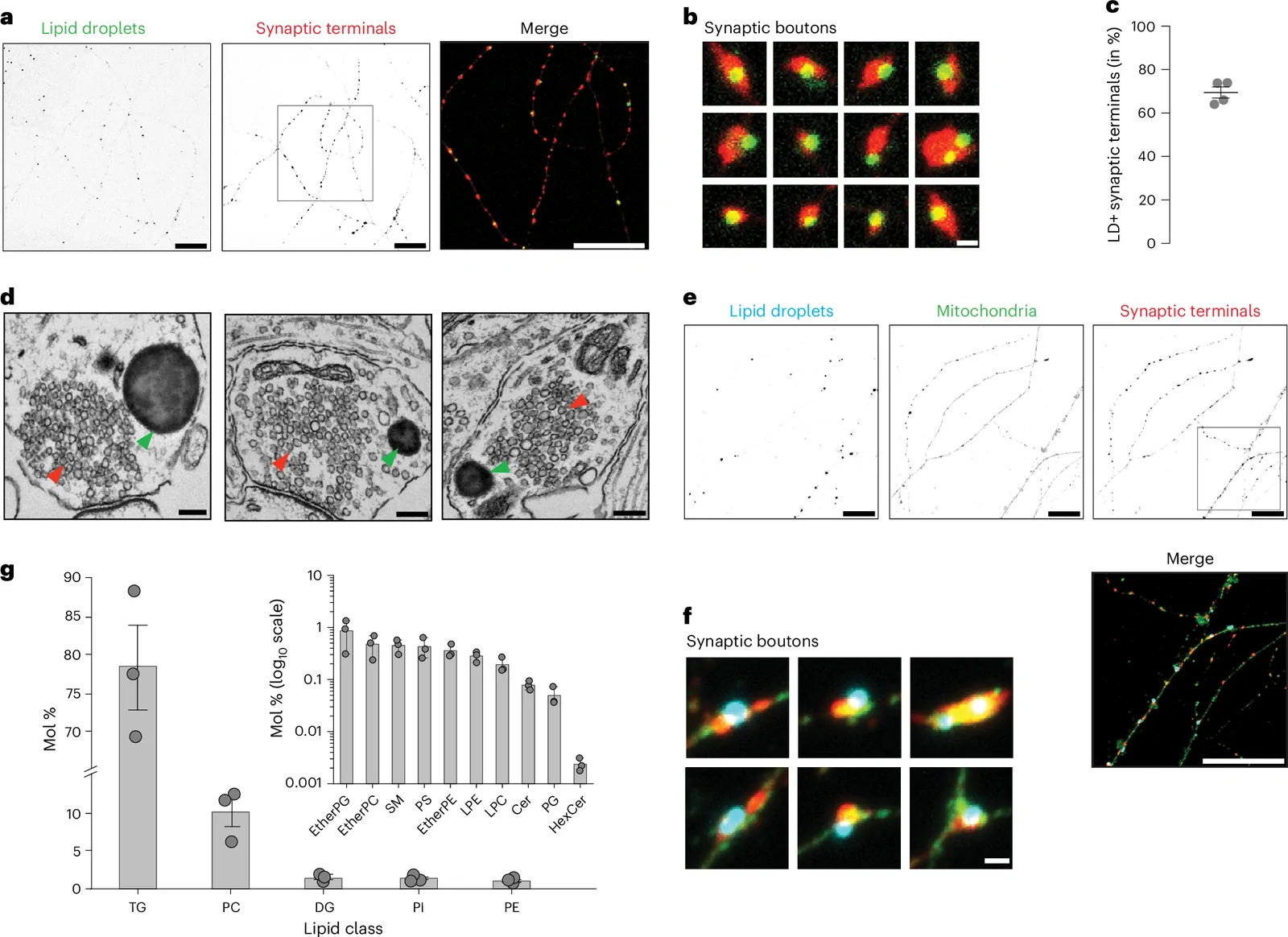
Inhibition of DDHD2 leads to LD accumulation at synaptic terminals. **a**, Confocal micrographs of KLH45-treated hippocampal neurons immunolabelled with synapsin (red, synaptic terminal) antibody and stained with BODIPY (green, LD marker). The boxed region is shown as a zoomed-in merged image. Scale bar, 20 µm. **b**, Zoomed-in images of 12 individual synaptic boutons from **a** showing LD localization. Scale bar, 1 µm. **c**, Quantification of synaptic terminals positive for BODIPY-stained LDs. Data are presented as means for regions of interest quantified from *n* = 4 independent experiments; error bars, s.e.m. **d**, Electron micrographs of KLH45-treated hippocampal neurons (18 days in vitro). LDs and SVs are indicated by green and red arrowheads, respectively. Scale bar, 200 nm. **e**, Confocal micrographs of KLH45-treated hippocampal neurons immunolabelled with TOMM20 (green, mitochondria) and synapsin (red, synaptic terminals) antibodies, and stained with monodansylpentane (MDH, AUTODOT) (cyan, LD marker). The boxed region is shown as a zoomed-in merged image in the lower panel. Scale bar, 20 µm. **f**, Zoomed-in images of six synaptic boutons from **e** showing localization of LDs and mitochondria at synaptic terminals. Scale bar, 1 µm. **g**, Lipid composition of purified LDs from cortical neurons, quantified (in mole %) using liquid chromatography–mass spectrometry. Minor lipids are displayed on a logarithmic scale in the inset. Standard lipid abbreviations are used. Data from three independent experiments are presented as means; error bars, s.e.m. PI, phosphatidylinositol; PE, phosphatidylethanolamine; PG, phosphatidylglycerol; SM, sphingomyelin; PS, phosphatidylserine; LPE, lysophosphatidylethanolamine; LPC, lysophosphatidylcholine; Cer, ceramide; HexCer, hexosylceramide. Source data

**Extended Data Fig. 3:**
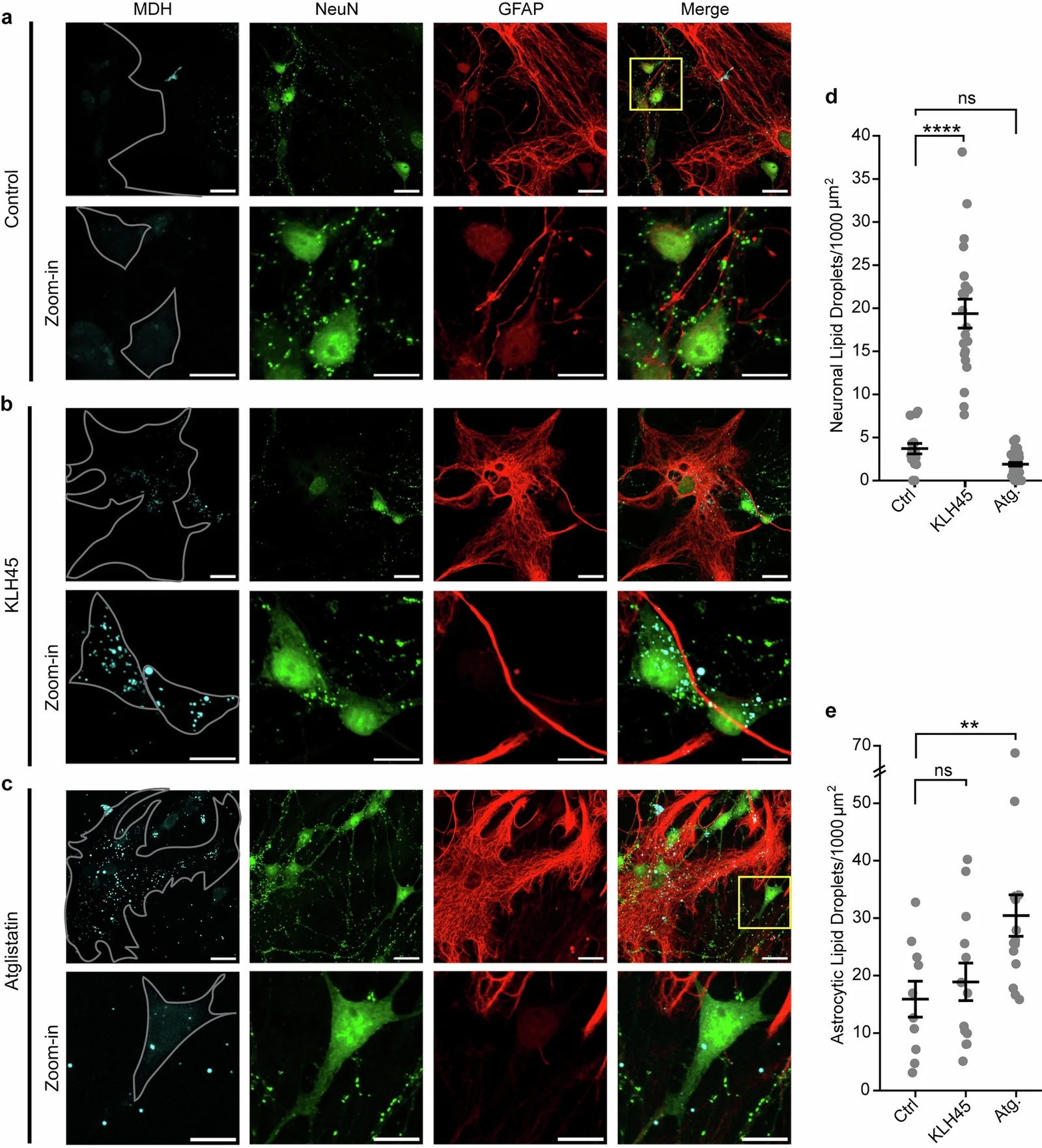
Effect of DDHD2 and ATGL lipase inhibition on lipid accumulation in neurons and astrocytes. **a**, Confocal micrographs of control neurons (green, NeuN) and astrocytes (red, GFAP) stained with MDH/AUTODOT (cyan, lipid droplets). Scale bar: 20 μm. **b**, Confocal micrographs of KLH45 (DDHD2 inhibitor)-treated neurons (NeuN) and astrocytes (GFAP) stained with MDH (lipid droplets). Scale bar: 20 μm. **c**, Confocal micrographs of atglistatin (ATGL inhibitor)-treated neurons (NeuN) and astrocytes (GFAP) stained with MDH (lipid droplets). Scale bar: 20 μm. Lower panels (in **a**-**c**) show zoomed-in views of the upper panels (marked with yellow boxes). Scale bars: 10 μm. **d**, Quantification of LD numbers in hippocampal neurons following KLH45 and atglistatin treatment. Data are presented as mean ± SE. *p*-values (ns, *p* = 0.33; ****p < 0.0001) were determined using one-way ANOVA followed by Tukey’s multiple comparison test with n = 16 for Ctrl, n = 21 for KLH45 and n = 39 for atglistatin. **e**, Quantification of LD numbers in astrocytes following KLH45 and atglistatin treatment. Data are presented as mean ± SE. *p*-values (ns, *p* = 0.83; **p = 0.01) were determined using one-way ANOVA followed by Tukey’s multiple comparison test with n = 10 for Ctrl, n = 13 for KLH45, and n = 15 for atglistatin. Source data

**Extended Data Fig. 4:**
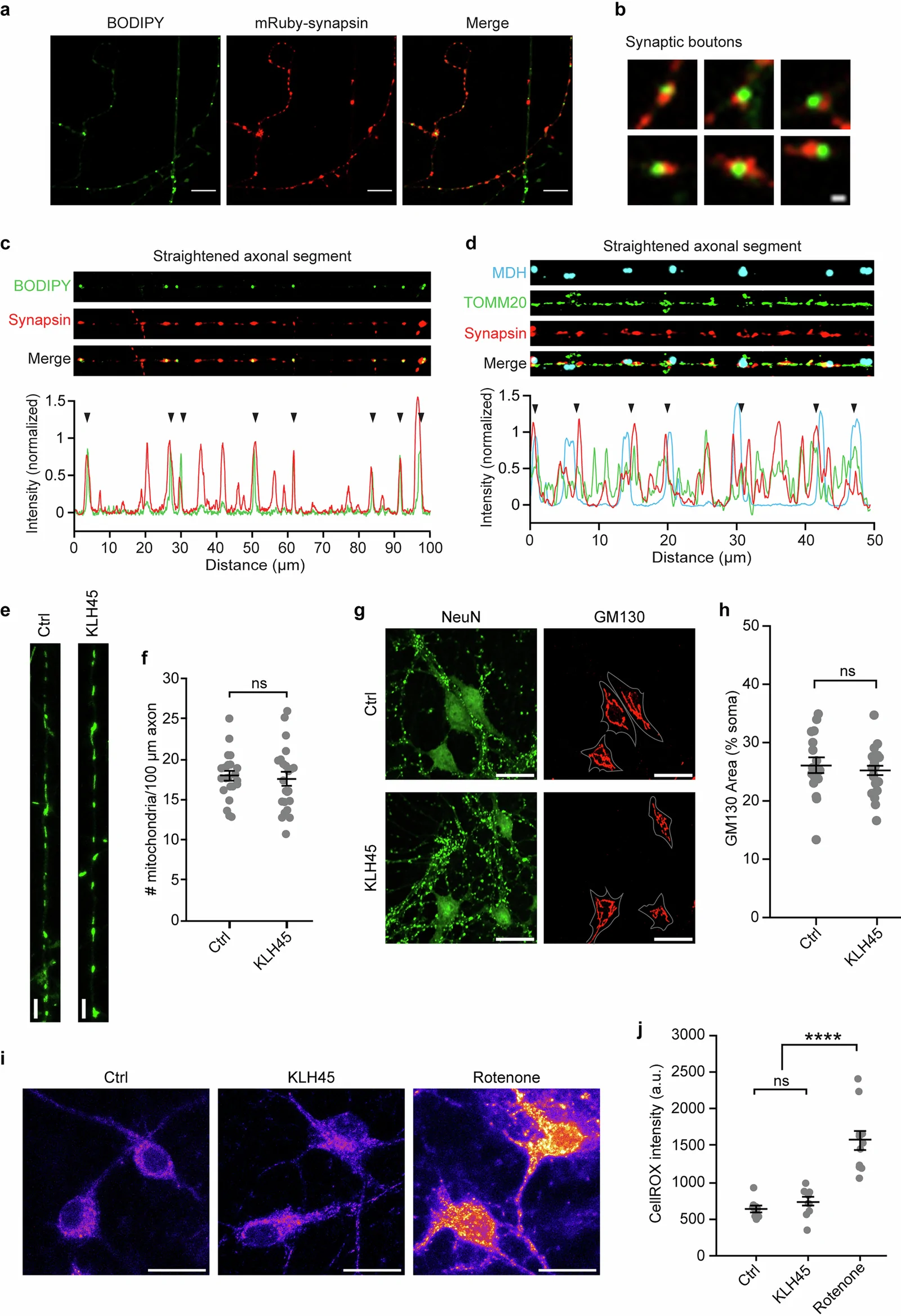
KLH45-induced LDs co-localize with mitochondria in axons without affecting mitochondrial or Golgi abundance. **a**, Confocal micrographs of KLH45-treated hippocampal neurons expressing mRuby-synapsin and stained with BODIPY to label LDs. Scale bar: 10 μm. **b**, Zoomed-in synaptic boutons from (**a**). Scale bar: 1 μm. **c**, Confocal micrographs of a straightened 100 µm long segment of hippocampal axon (from Fig. 2a) immunolabeled with anti-synapsin antibody and stained with BODIPY. Merged image and corresponding line intensity profile show repeated overlapping peaks (black arrowheads) of BODIPY (green, LDs) and synapsin (red, synaptic boutons). **d**, Confocal micrographs of a straightened 50 µm long segment of hippocampal axon (from Fig. 2e) stained with MDH (cyan, LDs) and immunolabeled with anti-TOMM20 (green, mitochondria) and anti-synapsin (red, synaptic boutons) antibodies. Merged image and corresponding line intensity profile show repeated MDH-positive puncta, adjacent to TOMM20 and synapsin peaks (black arrowheads). **e**, Confocal micrographs of straightened axonal segments from control and KLH45-treated neurons stained with MitoTracker Green. Scale Bar: 5 μm. **f**, Quantification of mitochondria per 100 μm axonal segment. Data are presented as mean ± SE. *p*-value (ns, *p* = 0.68) was determined using unpaired samples two-tailed *t*-test with n = 24 for Ctrl and n = 23 for KLH45 treated conditions. **g**, Confocal micrographs of untreated control and KLH45-treated hippocampal neurons immunostained with NeuN (green, neuron) and GM130 (red, cis-Golgi) antibodies. Scale bar: 20 µm. **h**, Quantification of cis-Golgi network area as a percentage of total neuronal area. Data are presented as mean ± SE. *p*-value (ns, *p* = 0.53) was determined using unpaired samples two-tailed *t*-test with n = 18 for Ctrl and n = 23 for KLH45 treated conditions. **i**, Confocal micrographs of untreated control, KLH45-treated, and Rotenone-treated hippocampal neurons stained with CellROX to assess ROS level. Rotenon, a mitochondrial complex-I inhibitor that induces ROS production, increases CellROX intensity. Scale bar: 20 μm. **j**, Quantification of CellROX fluorescence intensity in the soma. Data are presented as mean ± SE. *p*-values (ns, *p* = 0.73, ****p < 0.0001) were determined using one-way ANOVA followed by Tukey’s multiple comparison test with n = 10 for Ctrl and KLH45, and n = 11 for Rotenone treated conditions. Source data

Mice treated with KLH45 (a selective DDHD2 inhibitor) over a period of 4 days show significant elevation of brain TGs, similar to changes observed upon genetic ablation of this enzyme^6^. In mixed hippocampal culture, KLH45 treatment for 24 h resulted in more than a fivefold increase in neuronal LDs, whereas astrocytes showed no significant increase in LD number. By contrast, inhibition of another adipose triglyceride lipase using Atglistatin led to twofold increased LDs in astrocytes but not in neurons (Extended Data Fig. 3a–e). To further determine the subcellular localization of the LDs in polarized neurons, we treated sparsely plated dissociated hippocampal neurons with KLH45 and immunolabelled synaptic terminals with a synapsin antibody and the neutral lipid stain BODIPY(493/503), a convenient marker of mature LDs^15^ (Fig. 2a,b). BODIPY staining of mRuby–synapsin-expressing KLH45-treated hippocampal neurons showed localization of LDs in close proximity to synapsin (Extended Data Fig. 4a,b). Quantification of BODIPY-positive synapsin puncta revealed that ~70% of synaptic terminals harbour LDs (Fig. 2c), demonstrating that most nerve terminals appear to be constantly consuming LD TGs. Electron-microscopy-based ultrastructural analysis confirmed the presence of electron-dense LDs close to SV clusters (Fig. 2d). We further verified that LDs typically appear at nerve terminals with resident mitochondria by triple staining of KLH45-treated neurons with BODIPY (LDs), anti-TOMM20 (mitochondria) and anti-synapsin (synaptic terminals) antibodies (Fig. 2e,f). The line intensity profile of a 50–100 µm stretch of axonal processes shows frequent peaks of BODIPY and TOMM20 at synaptic terminals (Extended Data Fig. 4c,d). As a control, KLH45 treatment did not alter the mitochondria density along axonal processes (Extended Data Fig. 4e,f). Given that knockdown of DDHD2 has been associated with cellular stress to neurons^16^, we assessed whether our prolonged KLH45 treatment paradigm induces cellular stress to neurons by examining *cis*-Golgi morphology (by GM130 immunostaining) and oxidative stress level (by CellROX staining). Notably, we observed no change in the cellular distribution of the *cis*-Golgi network (Extended Data Fig. 4g,h) or CellROX intensity (Extended Data Fig. 4i,j).

**Extended Data Fig. 5:**
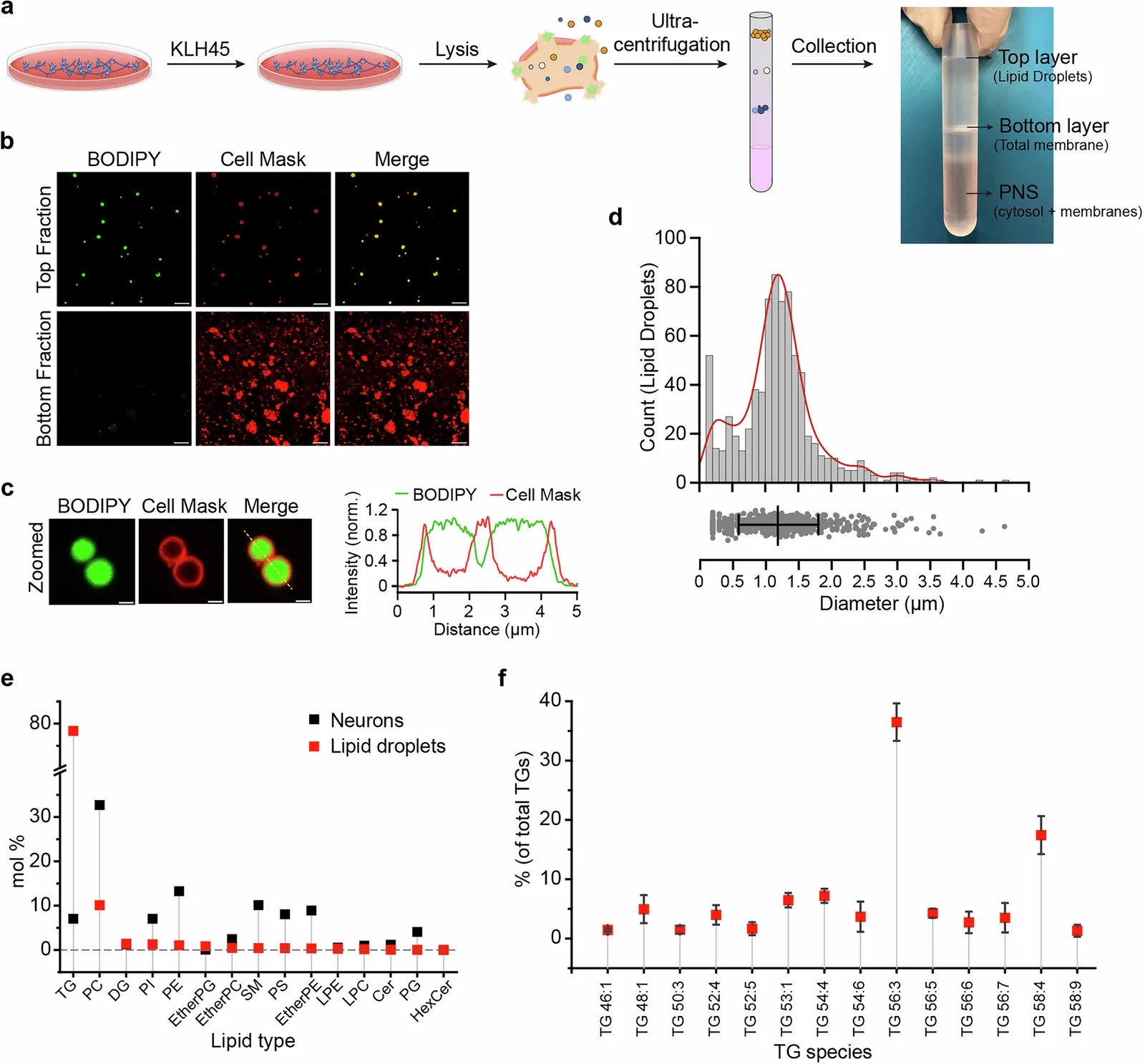
Purification and lipid profiling of KLH45-induced neuronal LDs. **a**, Schematic illustration of the experimental workflow used to induce LD formation in cortical neurons and isolate them via density-based floatation using sucrose step-gradient. Floating LDs were collected from the top layer of sucrose-free MEPS buffer [see detailed method in *Kumar* et*.* al*. 2023*]. Bottom layer contained relatively denser bi-layered membranous organelles with aqueous cores. **b**, Confocal micrographs of purified LDs (top layer) and membranous organelles (bottom layer) stained with BODIPY (green, neutral lipid dye) and CellMask (red, amphipathic membrane dye). Absence of CellMask-positive, and BODIPY-negative structures in top-fraction confirms the purity of isolated LDs. Additionally, lack of detectable BODIPY-positive structures in the bottom fraction confirms minimal loss of LDs during floatation process. Scale bar: 10 µm. **c**, Zoomed-in confocal micrographs and intensity profile (along yellow line) of BODIPY- and CellMask-stained LDs show a core of neutral lipids enclosed by a phospholipid layer. Scale bar: 1 µm. **d**, Quantification of LD diameters based on confocal micrographs of BODIPY-stained LDs. Data are presented as mean ± SD. LD diameter was estimated to be ~1.2 µm based on measurements from n = 761 droplets. **e**, Relative abundance (mole %) of lipid species in purified LDs compared to whole neuron lipid composition after KLH45-induction. Mean values for each lipid class were calculated from three independent experiments. **f**, Comparative abundance (in % of total TGs) of triglyceride species categorized by fatty acid chain length and degree of saturation. Data are presented as mean ± SE from three independent experiments. Source data

We carried out a biochemical purification of KLH45-induced LDs in our neuronal culture using a sucrose density gradient (Extended Data Fig. 5a) to further characterize their lipid content. To ascertain the purity of the isolated LD fraction, we stained the top and dense bottom membrane layers, respectively, resulting from centrifugation with BODIPY and CellMask (an amphipathic membrane dye)^17^. In the LDs containing the top layer, the only CellMask labelling was represented by the outer profile of the BODIPY-positive core, whose average diameter was 1.2 µm (Extended Data Fig. 5b–d). Quantitative liquid chromatography–mass spectrometry analysis of these LDs showed that they consisted of 78.36 ± 4.52 mol% TGs, 1.42 ± 0.22 mol% diacylglycerol (DG) and 10.1 ± 1.63 mol% phosphatidylcholine (PC) (Fig. 2g and Extended Data Fig. 5e,f). We also identified a lower abundance of other lipid classes in LDs and compared them with total cellular lipids (Fig. 2g and Extended Data Fig. 5e). A comprehensive stepwise method for purifying and performing lipidomic profiling of the neuronal LDs can be accessed elsewhere^18^.

**Extended Data Fig. 6:**
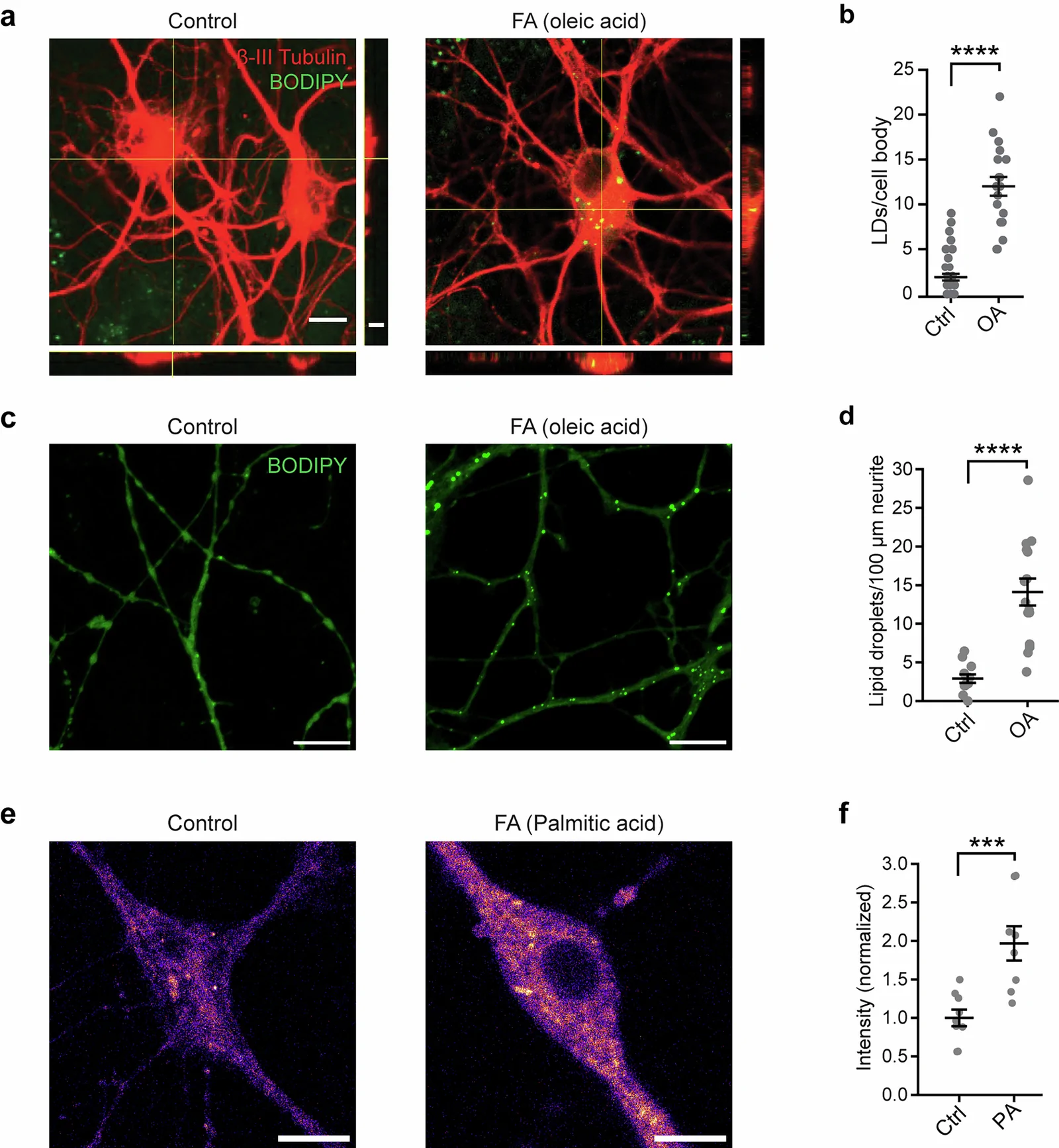
Albumin-complexed fatty acids induce lipid droplet formation in neurons. **a**, Confocal micrographs of soma from control and oleic acid (OA, complexed with bovine serum albumin)-treated hippocampal neurons. Neurons were immunostained with anti-β-III tubulin antibody (red, neuron) and stained with BODIPY (green, LD). Scale bar: 10 µm. Orthogonal sections along the yellow lines are shown to indicate globular LDs in neuronal cytosol. Scale bar: 5 µm. **b**, Quantification of LDs in the soma of control and OA-fed hippocampal neurons. Data are presented as mean ± SE. *p*-value (****p < 0.0001) was determined using unpaired samples two-tailed *t*-test with n = 21 for Ctrl and n = 19 for OA treated conditions. **c**, Confocal micrographs of axonal processes of control and OA-treated hippocampal neurons, stained with BODIPY and imaged live to visualize lipid droplets. Scale bar: 10 µm. **d**, Quantification of LDs along axonal processes in control and OA-treated neurons. Data are presented as mean ± SE. *p*-value (****p < 0.0001) was determined using unpaired samples two-tailed *t*-test with n = 12 for Ctrl and n = 15 for OA treated conditions. **e**, Confocal micrographs of BODIPY-stained soma of control and palmitic acid (PA, complexed with bovine serum albumin)-treated hippocampal neurons. Scale bar: 10 µm. **f**, Quantification of fluorescent intensities in the soma of control and PA-treated neurons. Data are presented as mean ± SE. *p*-value (***p = 0.0006) was determined using unpaired samples two-tailed *t*-test with n = 9 for Ctrl and n = 8 for PA treated conditions. Source data

These data demonstrate that under steady-state metabolic conditions, TGs are continuously being synthesized and consumed, as blocking DDHD2 activity shifts the balance to a net accumulation of LDs. We tested an alternative approach to tilt the balance towards LD accumulation: providing excess FAs to the growth medium. BODIPY staining in neurons, whose culture media was supplemented with excess mono-unsaturated FA (oleic acid) for 24 h, demonstrated that increasing the availability of FAs results in a net accumulation of LDs in soma (Extended Data Fig. 6a,b) and neuronal processes (Extended Data Fig. 6c,d). Similarly, supplementation with a saturated FA (palmitic acid) resulted in enhanced BODIPY staining in neurons (Extended Data Fig. 6e,f), confirming increased LD formation. These experiments together suggest that active DDHD2 at synapses prevents excessive LD accumulation by maintaining a dynamic balance between TG synthesis and consumption.

### Metabolic demand in neurons triggers TG mobilization from LDs

**Fig. 3:**
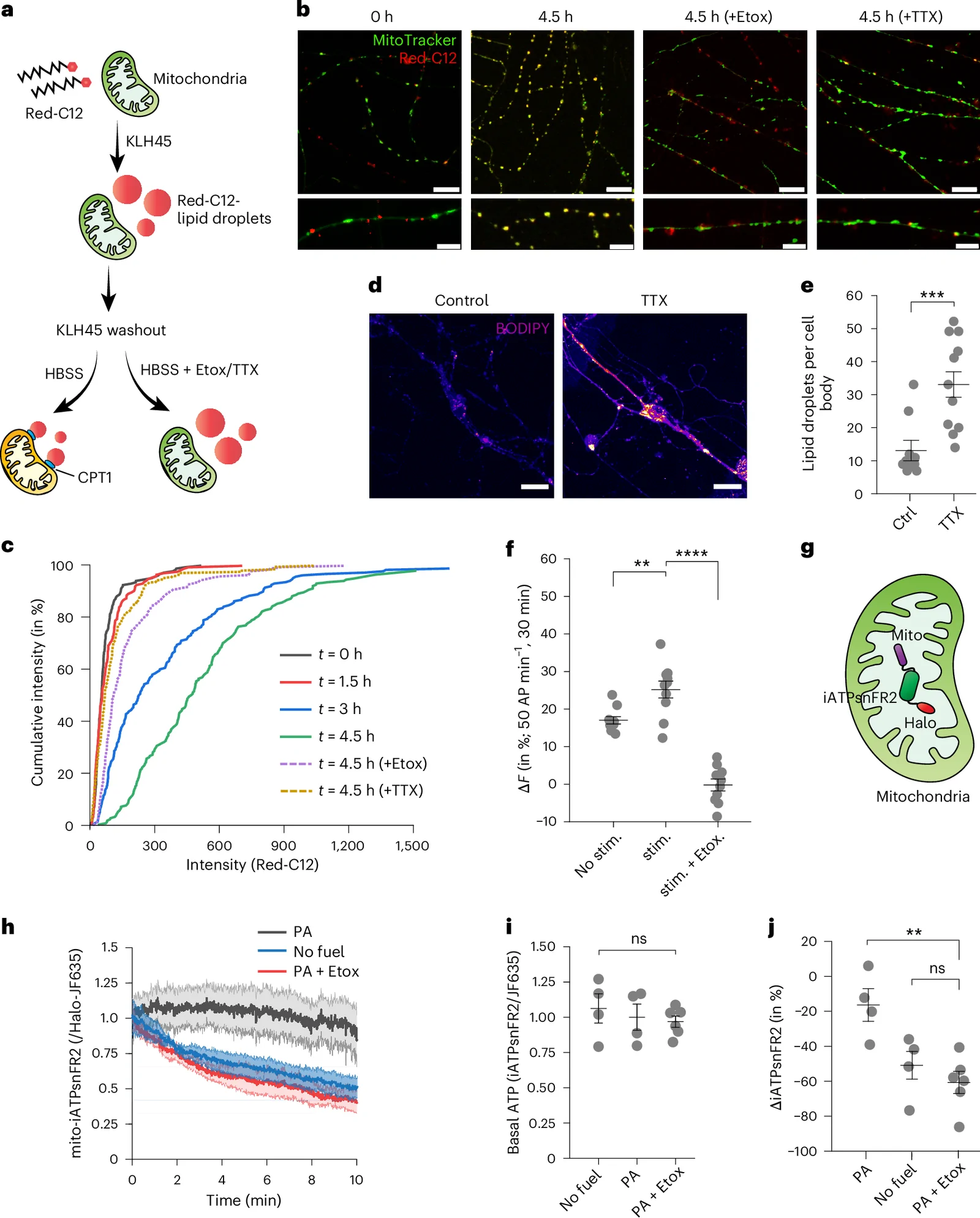
Synaptic activity and CPT1 facilitate FA transfer to the mitochondrial matrix to sustain ATP levels in neurons. **a**, Schematic representation depicting the accumulation of Red-C12 (BODIPY 558/568 C12 FA) in LDs following KLH45 treatment, and subsequent washout with or without etomoxir (Etox). CPT1 and CPT2 (CPT2 not shown) on mitochondrial membranes facilitate the transfer of Red-C12 to the mitochondrial matrix in HBSS media. **b**, Confocal micrographs showing transfer of Red-C12 to mitochondria within 4.5 h in the absence of external glucose. The presence of etomoxir or TTX in HBSS media inhibits Red-C12 transfer to mitochondria. Scale bars, 10 µm (upper panels) and 5 µm (lower panels). **c**, Cumulative per cent transfer of Red-C12 to mitochondria at 0 h, 1.5 h, 3 h and 4.5 h after HBSS media change. Dotted lines indicate reduced Red-C12 transfer when etomoxir or TTX was present in HBSS. **d**, Confocal micrographs of control and TTX-treated (72 h) hippocampal neurons stained with BODIPY. Scale bar, 20 µm. **e**, Quantification of LD number in the soma of control and TTX-treated neurons. Data are presented as means; error bars, s.e.m. *P* value (***P = 0.001) was determined using an unpaired samples two-tailed *t*-test with *n* = 10 for Ctrl and *n* = 12 for TTX. **f**, Quantification of Red-C12 colocalization with MitoTracker Green in neurons subjected to electrical stimulation (50 AP min^−1^ for 30 min) compared with unstimulated controls. AP, action potential; stim., stimulated; Etox., etomoxir. Etomoxir-treated neurons show reduced Red-C12 colocalization. Data are presented as means; error bars, s.e.m. *P* values (**P = 0.0053; ****P ≤ 0.0001) were determined using one-way ANOVA with Tukey’s multiple comparison test for *n* = 10 in each condition. **g**, Schematic representation of the mitochondrial ATP sensor (iATPsnFR2) with a C-terminal Halo tag, targeted to the mitochondrial matrix by four repeats of the amino-terminal leader sequence, mito/COX8. **h**, Relative ATP levels (normalized with JF635 for expression) in mito-iATPsnFR2-Halo-expressing hippocampal neurons under different metabolic conditions: no external fuel, palmitic acid (PA) supplementation or CPT1 inhibition (by etomoxir) to PA-induced neurons. **i**, Basal intensity of iATPsnFR2 under conditions described in **h**. **j**, Relative change (%) in ATP levels after 10 min of perfusion with the media described in **h**. Data in **h**–**j** are presented as means; error bars, s.e.m. *P* values (ns, *P* > 0.05; **P = 0.006) in **i** and **j** were determined using one-way ANOVA with Tukey’s multiple comparison test. Sample sizes in **h**–**j** were *n* = 4 for no fuel and PA conditions, and *n* = 6 for PA + Etox conditions. Source data

**Extended Data Fig. 7:**
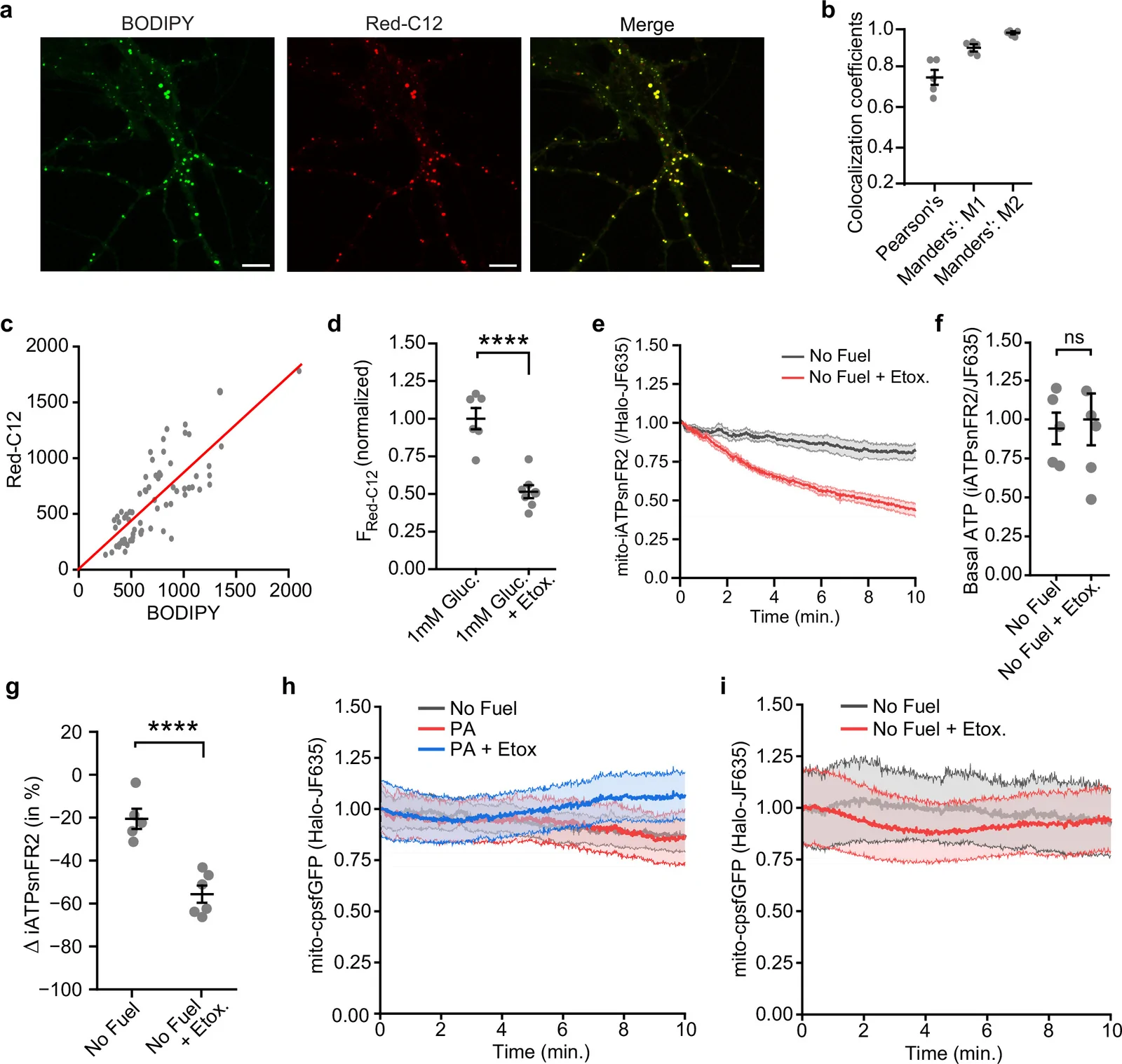
Lipid droplet-derived fatty acids sustain mitochondrial ATP levels in neurons. **a**, Confocal micrographs showing colocalization of BODIPY493/503 labeled globular structures (LDs) with Red-C12, confirming the storage of exogenous fatty acids within LD core. Scale bar: 10 µm. **b**, Quantification of Pearson’s colocalization coefficient from confocal images in (**a**). Data are presented as mean ± SE for N = 5. M1: fraction of Red-C12 overlapping with BODIPY, M2: fraction of BODIPY overlapping with Red-C12. **c**, Correlation between fluorescence intensities of BODIPY 493/503 and Red-C12 measured across 70 LDs (Pearson’s r: 0.79, R^2^: 0.9). **d**, Comparison of Red-C12 colocalization with MitoTracker Green in neurons cultured in feeding media containing 1 mM glucose or 1 mM glucose with etomoxir. Data are presented as mean ± SE. *p*-value (****p < 0.0001) was determined using unpaired samples two-tailed *t*-test with N = 6 for Glucose and N = 7 for Glucose + Etomoxir conditions. **e**, ATP levels (normalized to JF635) in mito-iATPsnFR2-Halo expressing hippocampal neurons after induction with KLH45 followed by fuel-deprivation compared to CPT1 inhibited (by etomoxir) conditions. **f**, Basal intensity of iATPsnFR2 prior to media perfusion as indicated in (**e**). **g**, Relative change in ATP levels after 10 minutes of perfusion with the media described in (**e**). Data in (**e**-**g**) are presented as mean ± SE. *p*-values (ns, *p* = 0.76; ****p = 0.0001) were determined using unpaired samples two-tailed *t*-test with N = 5 for No Fuel and N = 6 for No Fuel + Etomoxir conditions. **h-i**, Fluorescence intensity of cpsfGFP (normalized to JF635) in mito-cpsfGFP-Halo expressing hippocampal neurons under conditions described in Fig. 3h and Extended Data Fig. 7e. Data are presented as mean ± SE. Samples sizes were N = 5 for No Fuel (**h**), N = 6 for PA, N = 6 for PA + Etomoxir, N = 4 for No Fuel (**i**), and N = 4 for No Fuel + Etomoxir treated conditions. Source data

In various tissues, TGs are stored in LDs to serve as an alternative fuel source when glucose availability is limited. Lipases are necessary for TG hydrolysis, converting them into FAs in the cytosol. Subsequently, these FA molecules are converted into fatty acyl-CoA and delivered to the mitochondrial matrix by the mitochondrial carnitine palmitoyl-transferases (CPTs) for oxidation^19^. To determine whether FAs derived from LDs in axons are delivered to axonal mitochondria, we used a pulse–chase strategy with FA covalently conjugated with BODIPY 558/568, Red-C12 (Fig. 3a). A 24 h pulse of Red-C12 was delivered to dissociated hippocampal neurons in the presence of KLH45 to facilitate its accumulation in LDs (Extended Data Fig. 7a–c), followed by removal of KLH45 during chase period. The amount of Red-C12 accumulated in mitochondria was assessed by measuring the fluorescence intensity of Red-C12 that overlapped with MitoTracker Green^20^. At 24 h after pulsing in Red-C12 during DDHD2 block, Red-C12 exhibited both diffuse axonal labelling and punctate distribution that were positive for LD markers (Extended Data Fig. 7a), with minimal colocalization with mitochondria (Fig. 3b, see 0 h in Fig. 3c). However, when neurons were shifted to KLH45-free Hanks’ balanced salt solution (HBSS) media, we observed a gradual transfer of Red-C12 into mitochondria during the chase period (Fig. 3b, see 1.5–4.5 h in Fig. 3c). By contrast, including the CPT1 inhibitor etomoxir during the chase period prevented transfer of Red-C12 to mitochondria (Fig. 3b,c). To determine whether Red-C12 is transferred to mitochondria in the presence of a physiological level of glucose, we pulsed Red-C12 in feeding media containing 1 mM glucose. Red-C12 colocalized with MitoTracker after 24 h in glucose; however, this colocalization was reduced to 50% in the presence of etomoxir (Extended Data Fig. 7d).

### Activity controls FA transfer from LDs to axonal mitochondria

We previously showed that electrical activity is tightly coupled to ATP synthesis in neuronal axons to meet the demands of nerve terminal function^21^ through both upregulation of glucose uptake^22^ and mitochondrial respiration^23^. To explore the potential impact of electrical activity on LD storage or usage, we treated dissociated neuronal cultures with the Na^+^ channel blocker tetrodotoxin (TTX) for 72 h and examined the LD content compared to untreated neurons using BODIPY. These experiments demonstrated that blocking chronic electrical activity leads to a significant accumulation of LDs, both in cell somas and in neuronal processes (Fig. 3d,e). This result implies that electrical activity modulates some combination of LD formation or consumption. To determine whether LD consumption might be modulated, we used our Red-C12 pulse–chase approach but blocked electrical activity during the chase period with TTX (after removal of the DDHD2 inhibitor) (Fig. 3b,c). To directly assess the dependency of FA transfer to mitochondria, we stimulated hippocampal neurons with bouts of 50 AP min^−1^ for 30 min. This significantly enhanced Red-C12 localization with mitochondria compared to unstimulated controls, whereas the colocalization was abolished in the presence of etomoxir (Fig. 3f). These experiments revealed that blocking electrical activity suppressed the CPT1-mediated transfer of FA into the mitochondrial matrix, demonstrating that consumption of LDs in axons is modulated by electrical activity. These findings also demonstrate that the abundance of LDs in neurons is determined by both the availability of FAs as well as electrical activity, which, in turn, modulates the consumption of LDs.

### LD-derived FAs support axonal ATP production through β-oxidation

Fatty acyl-CoA chains transferred to the mitochondrial matrix undergo a cyclic process to generate acetyl-CoA, which subsequently enters the tricarboxylic cycle to produce ATP. We made use of a newly developed, genetically encoded ATP sensor with a large dynamic range targeted to the mitochondrial matrix, mito-iATPSnFR2 (ref. ^24^), to examine ATP levels in mitochondria (Fig. 3g). The ATP sensor (normalized with JF635 for expression) in primary neurons was used to quantify ATP production in the mitochondrial matrix under different fuel conditions. Following acute removal of glucose, axonal mitochondrial ATP declined slowly to 50.1 ± 9.2% for the next 10 min, as expected, given that pyruvate production by glycolysis should be largely absent. However, in neurons that had been pre-fed with palmitic acid for 4 h, mitochondrial ATP levels declined only 16.37 ± 8.08% over the same period in the complete absence of glucose. By contrast, if the CPT1 inhibitor etomoxir was applied at the same time as the glucose was removed, the decline in mitochondrial ATP levels in palmitic acid pre-fed neurons was 61.9 ± 5.8% (Fig. 3h,j). Basal ATP levels in all conditions were the same before replacing the fuel (Fig. 3i). Similar results were obtained in neurons in which LDs were induced by blocking DDHD2 activity with KLH45 for 24 h. When glucose and KLH45 are removed, ATP levels are sustained up to 81.7 ± 4.08% of the initial value, but including etomoxir under these conditions leads ATP to decline rapidly to 43.5 ± 3.9% over a 10-min period (Extended Data Fig. 7e–g). Furthermore, in neurons expressing a control sensor with similar pH sensitivity to mito-iATPSnFR2 but insensitive to ATP (mito-cpsfGFP-Halo), the fluorescent intensity remained unchanged across all conditions (Extended Data Fig. 7h,i), indicating that the observed changes in the iATPsnFR2 signal were specific to ATP levels and not influenced by intrinsic factors such as pH fluctuations. These experiments demonstrate that axonal mitochondria can sustain mitochondrial ATP production using FAs derived from LDs in the complete absence of glucose.

### LDs can support vesicle recycling in hypoglycaemic conditions

**Fig. 4:**
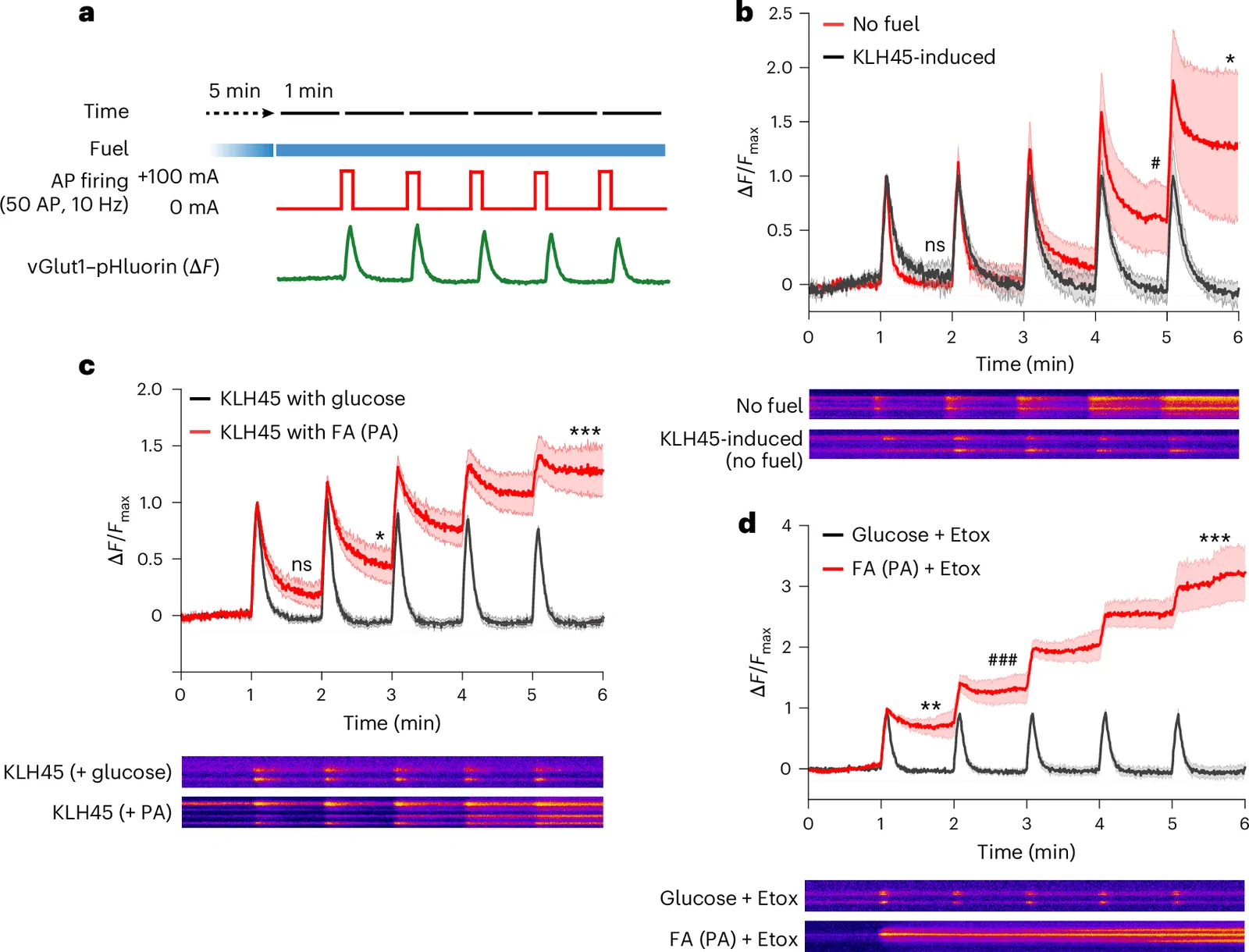
LD-induced neurons exhibit improved synaptic endurance. **a**, Schematic representation of the experimental paradigm (adapted from a previous publication^25^): dissociated hippocampal neurons expressing vGlut1–pHluorin were perfused with indicated media for 5 min (unless otherwise specified), followed by AP firing (+100 mA, 50 AP, 10 Hz) at 1 min intervals. The fluorescence intensity shift of pHluorin (∆*F*) at the synaptic boutons (regions of interest, 12–18 µm²), reflecting exocytosis and endocytosis of SVs, was recorded and normalized to the peak response of the first AP firing. **b**, Fluorescence intensity traces at synaptic boutons following periodic AP firing in the absence of any external fuel (labelled as ‘No fuel’) versus TG-loaded hippocampal neurons after KLH45 washout (labelled as ‘KLH45-induced’). Unlike the no-fuel condition, TG-loaded neurons sustain five rounds of SV recycling even in the absence of external fuel. Sample sizes were *n* = 4 for no fuel and *n* = 8 for KLH45-induced conditions. *P* values (ns, *P* = 0.974; ^#^*P* = 0.012; *P = 0.014) were determined using an unpaired samples two-tailed *t*-test. **c**, Fluorescence intensity traces at synaptic boutons following periodic AP firing in glucose or PA-fed hippocampal neurons in the presence of KLH45. Sample sizes were *n* = 6 for KLH45 with glucose and *n* = 7 for KLH45 with FA (PA) conditions. *P* values (ns, *P* = 0.098; *P = 0.0127; ***P = 0.0002) were determined using an unpaired samples two-tailed *t*-test. **d**, Fluorescence intensity traces at synaptic boutons following periodic AP firing in glucose or PA-fed hippocampal neurons in the presence of etomoxir. Sample sizes were *n* = 5 for both glucose + Etox and FA (PA) + Etox conditions. *P* values (**P = 0.007; ^###^*P* = 0.0007; ***P = 0.0001) were determined using an unpaired samples two-tailed *t*-test. Data in **b**–**d** are presented as means; error bars, s.e.m. Representative kymographs depicting vGlut1–pHluorin fluorescence intensity changes at the synaptic boutons are shown below their respective traces. Asterisk (*) and hash (#) symbols are used to distinguish between different groups of statistically significant comparison. Source data

**Extended Data Fig. 8:**
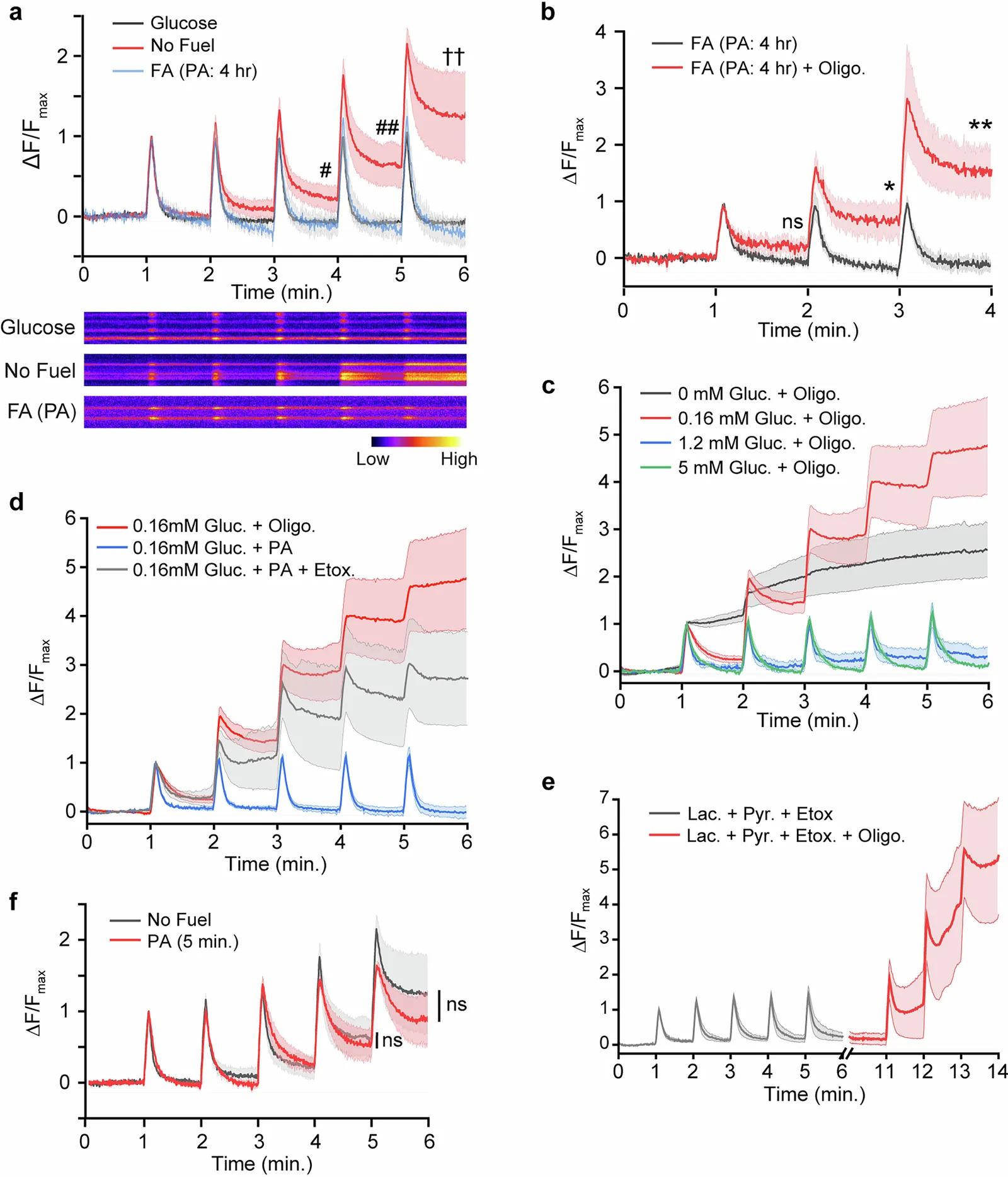
Palmitic acid (PA) supports synaptic vesicle recycling in hypometabolic condition. **a**, Fluorescence intensity traces (normalized to first peak) of vGlut1-pHluorin at synaptic boutons of dissociated hippocampal neurons following periodic action potential firing (as in Fig. 4a) under three conditions: absence of external fuel (N = 9), presence of 5 mM glucose (N = 5), or pre-fed and maintained with PA (N = 5). Lower panels show representative kymographs of synaptic boutons under these conditions. **b**, Fluorescence intensity traces of vGlut-pHluorin at synaptic boutons in PA pre-fed or following oligomycin treatment in PA-fed hippocampal neurons. Sample sizes were N = 5 for both PA and PA + Oligo. treated conditions. **c**, Fluorescence intensity traces of vGlut1-pHluorin at synaptic boutons of dissociated hippocampal neurons maintained with 0, 0.16, 1.2, and 5 mM glucose, in presence of 2 µM oligomycin. Sample sizes were N = 6 for 0 mM Glucose + Oligo, N = 5 for 0.16 mM Glucose + Oligo, N = 4 for 1.2 mM Glucose + Oligo and N = 5 for 5 mM Glucose + Oligo conditions. **d**, Fluorescence intensity traces of vGlut1-pHluorin at synaptic boutons of hippocampal neurons maintained with following media: subphysiological 0.16 mM glucose + oligomycin (N = 5), pre-fed and maintained with PA + 0.16 mM glucose (N = 6), or pre-fed and maintained with PA + 0.16 mM glucose + etomoxir (N = 6). **e**, Synaptic vGlut1-pHluorin traces in lactate and pyruvate in presence of etomoxir, or lactate and pyruvate with etomoxir and oligomycin. Sample size for each condition was N = 5. **f**, Fluorescence intensity traces of vGlut-pHluorin at synaptic boutons after periodic AP firing in acutely PA-fed neurons (5 min.) compared to no fuel conditions. Sample size for each condition was N = 5. Data (in **a**-**f**) are presented as mean ± SE. *p*-values (ns, #*p* = 0.063; ##*p* = 0.01; ††*p* = 0.01; ns, *p* = 0.25; *p = 0.04; **p = 0.01) were determined using unpaired samples two-tailed *t*-test. Source data

Our data demonstrate that axonal DDHD2 lipase activity facilitates the release of FAs from LDs, fuelling mitochondrial ATP production. A critical question is whether the ATP produced from LD-derived FAs can support synaptic function. To test this idea, we used endocytic retrieval of vGlut1–pHluorin at synaptic terminals as a measure of metabolic endurance^25^. In the absence of an oxidizable carbon source, we previously showed that pHluorin-tagged SV proteins fail to recycle, instead accumulating on the plasma membrane or in an alkaline compartment^21,22^. Here, we deployed a more stringent synaptic endurance assay consisting of repeated bursts of 50 action potentials fired at 1-min intervals (Fig. 4a). Under sufficient fuel conditions (5 mM glucose), nerve terminals sustain SV recycling through at least five such rounds of stimulation; however, when glucose is removed, vesicle recycling fails within the first two to five rounds (Extended Data Fig. 8a). Notably, in neurons that were induced to allow LD accumulation, either by incubating neurons in KLH45 (Fig. 4b) or by including excess FAs (Extended Data Fig. 8a), SV recycling is sustained through multiple rounds of stimulation in the absence of glucose when KLH45 is not present. In control neurons running on glucose, KLH45 has no impact (Fig. 4c) and, as would be expected from a fuel that requires the electron transport chain, incubation with oligomycin completely arrests SV recycling, which depends on FAs (Extended Data Fig. 8b). These data strongly support the idea that FAs derived from LDs can serve as an alternate fuel. We found that SV recycling could be sustained even in the absence of mitochondrial ATP production, provided glucose levels were sufficiently high (green trace in Extended Data Fig. 8c). Under *F*_0_–*F*_1_ ATPase inhibition, as glucose levels are reduced from 1.2 mM to 0 mM, the SV recycling gradually ceased (Extended Data Fig. 8c). Notably, neurons exposed to 0.16 mM glucose showed significant impairment in SV recycling, indicating that sub-physiological glucose levels are insufficient to fully support synaptic function without a contribution from mitochondria. Under the same conditions, when neurons were pre-fed with palmitic acid, SV recycling was sustained, but is now dependent on the functionality of the mitochondrial FA import machinery (Extended Data Fig. 8d). SV recycling driven by lactate and pyruvate-fuelled mitochondrial ATP production remain intact even when CPT1 is inhibited (Extended Data Fig. 8e), demonstrating that on these time scales, etomoxir is not significantly impairing mitochondrial function.

**Extended Data Fig. 9:**
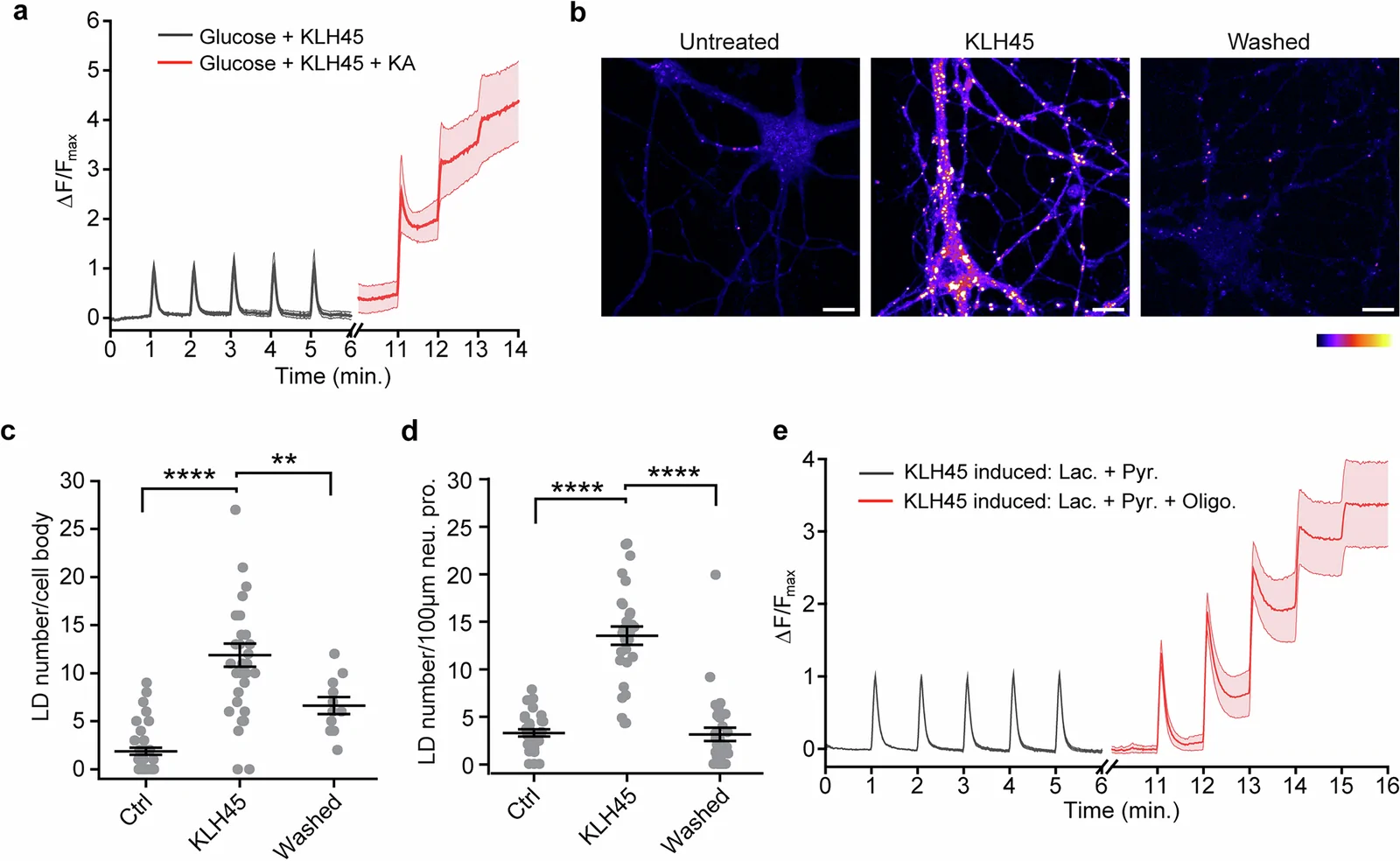
Restoration of DDHD2 activity reverses LD abundance and mitochondrial function. **a**, Normalized fluorescence intensity traces of vGlut1-pHluorin at synaptic boutons (as in Fig. 4a) of KLH45-treated (24 hrs.) hippocampal neurons in presence of 5 mM glucose and KLH45, followed by glucose, KLH45 and 10 µM koningic acid (KA, GAPDH inhibitor). Data are presented as mean ± SE for N = 5. **b**, Representative fluorescence images of BODIPY-stained hippocampal neurons showing untreated control, KLH45-treated, and 48-hour post-washout neurons. Scale bar: 10 µm. **c-d**, Quantification of LDs in the soma (**c**) and processes (**d**) from conditions shown in (**b**). Data are presented as mean ± SE. *p*-values (**p = 0.006, ****p < 0.0001) were determined using one-way ANOVA with Tukey’s multiple comparison test. Sample sizes in (**c**) were n = 40 for Ctrl, n = 33 for KLH45 and n = 11 for washed conditions. In (**d**), sample sizes were n = 29 for both Ctrl and KLH45, and n = 31 for washed condition. **e**, Normalized fluorescence intensity traces of vGlut1-pHluorin at synaptic terminals of KLH45-treated neurons (24 hrs.), maintained with 1.25 mM each of lactate and pyruvate, or lactate and pyruvate with oligomycin. The data are presented as mean ± SE for N = 5. Source data

Consistent with the need to assemble LDs, brief (5 min) incubation of neurons with extracellular FAs fail to sustain SV recycling in the absence of glucose (Extended Data Fig. 8f). As a control, neurons treated with KLH45 for 24 h could perform SV recycling in glucose, but obstructing glycolysis by GAPDH inhibition (using koningic acid) led to SV recycling failure (Extended Data Fig. 9a). These data indicate that pre-feeding neurons with FAs fuels ATP production by lipolysis of LD resident TGs. In agreement with these functional assays, with resumption of DDHD2 activity after KLH45 washout, the LD numbers are reduced both in the soma and axons (Extended Data Fig. 9b–d). Furthermore, the mitochondrial ability to generate ATP through lactate and pyruvate-fuelled oxidative phosphorylation was also efficiently restored, although it remained sensitive to oligomycin (Extended Data Fig. 9e).

**Extended Data Fig. 10:**
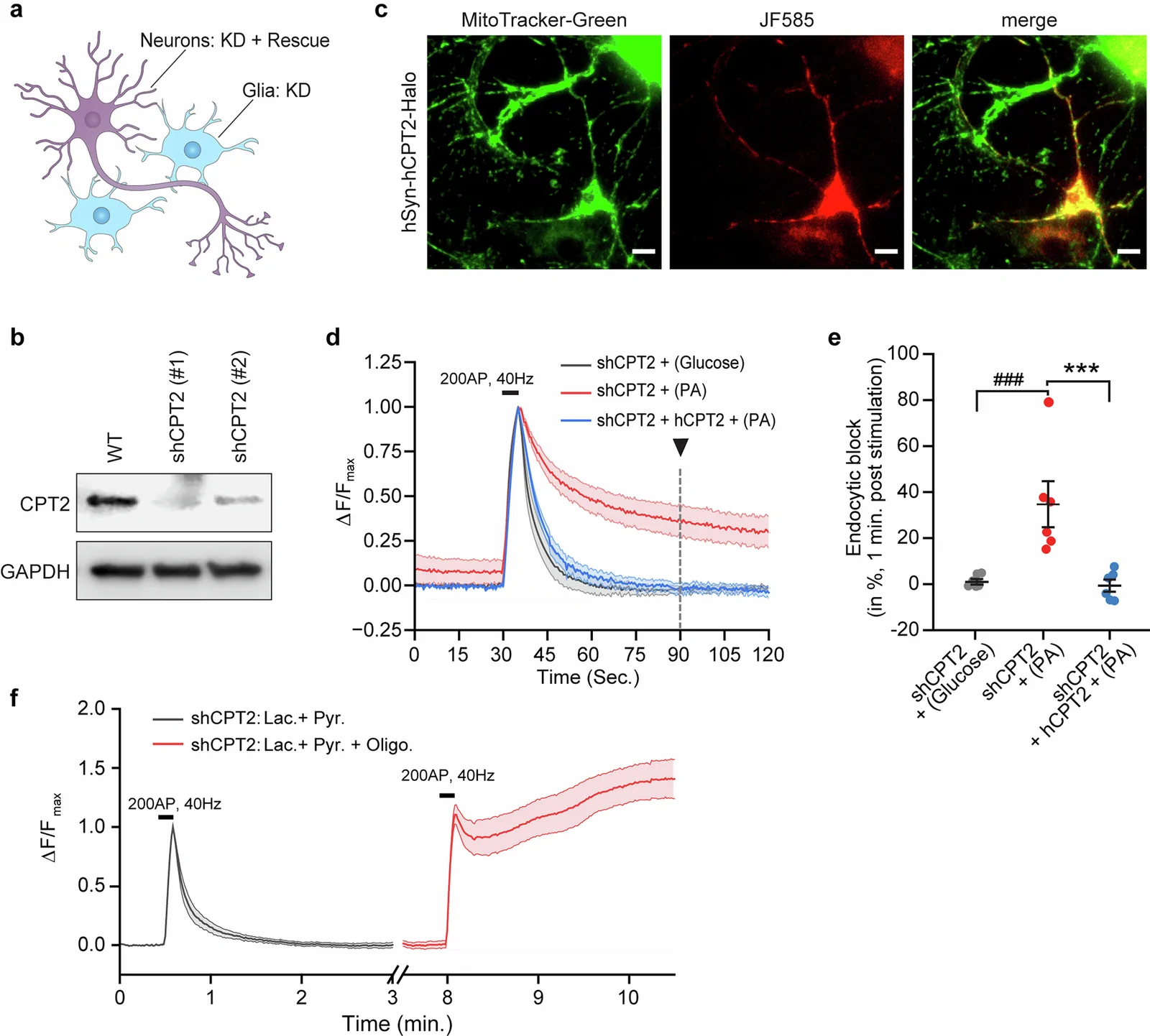
CPT2 facilitates fueling of synaptic vesicle recycling independent of astrocytic contributions. **a**, Schematic illustration of lenti-virus mediated knockdown (KD) of CPT2 in both glial cells and hippocampal neurons, with neuron-specific rescue achieved via overexpression of shRNA-resistant CPT2 under the control of human synapsin promotor. **b**, Western blot validation of CPT2 knockdown using two potential shRNAs. shCPT2 (#2) is used in subsequent experiments. Blot of GAPDH ensures equal loading of total proteins. **c**, Fluorescence micrographs showing colocalization of Halo-tagged human CPT2 (labeled with JF585, red) with MitoTracker (mitochondria, green) in hippocampal neurons, confirming mitochondrial targeting of CPT2-Halo. Scale bar: 20 µm. **d**, Normalized fluorescence intensity traces of vGlut1-pHluorin after 200AP firing to CPT2 depleted hippocampal neurons show reduced endocytosis in PA-fed compared to glucose-fed condition. Expression of shRNA resistant human CPT2 in neurons restored endocytosis. Data are presented as mean ± SE with N = 8 for shCPT2 + Glucose, N = 6 for shCPT2 + PA and N = 6 for shCPT2 + hCPT2 + PA. **e**, Quantification of endocytic block (in %) measured 60 seconds after AP firing, indicated by black arrowhead in (**d**). Data are presented as mean ± SE. *p*-values (###*p* = 0.0006, ***p = 0.0007) were determined using one-way ANOVA with Tukey’s multiple comparison test for the sample sizes as in (**d**). **f**, Normalized fluorescence intensity of vGlut1-pHluorin measured in CPT2-depleted hippocampal neurons electrically stimulated (as in d) in presence of 1.25 mM each lactate and pyruvate. CPT2 depletion did not impair lactate and pyruvate fueled endocytosis, whereas ATP synthase inhibition by oligomycin blocked post-stimulation endocytosis. Data are presented as mean ± SE with N = 7 for both conditions. Source data

To rule out the possibility that the FA-driven improvement in SV recycling may have been caused by metabolites arising from glial cells in the same dish, we used lentiviral delivery to transduce all cells in the dish, driving the expression of an shRNA targeting CPT2 (Extended Data Fig. 10a,b), which led to SV recycling failure even in the presence of LDs (red trace of Extended Data Fig. 10d), but showed that rescuing by expression of an shRNA-insensitive variant of CPT2 exclusively to neurons (Extended Data Fig. 10c) completely restored (−0.63 ± 2.3%) SV recycling (Extended Data Fig. 10d,e). To rule out any negative effect of CPT2 depletion on synaptic mitochondria, we performed an SV endurance assay in the presence of lactate and pyruvate. These neurons exhibited complete SV recycling, indicating that the mitochondrial OXPHOS function remained intact despite CPT2 depletion (Extended Data Fig. 10f). Taken together, these data demonstrate that presynaptic function can be sustained purely from axonal LD-derived FAs.

### Acute blockade of CPT1 or DDHD2 lipase induces torpor in mice

**Fig. 5:**
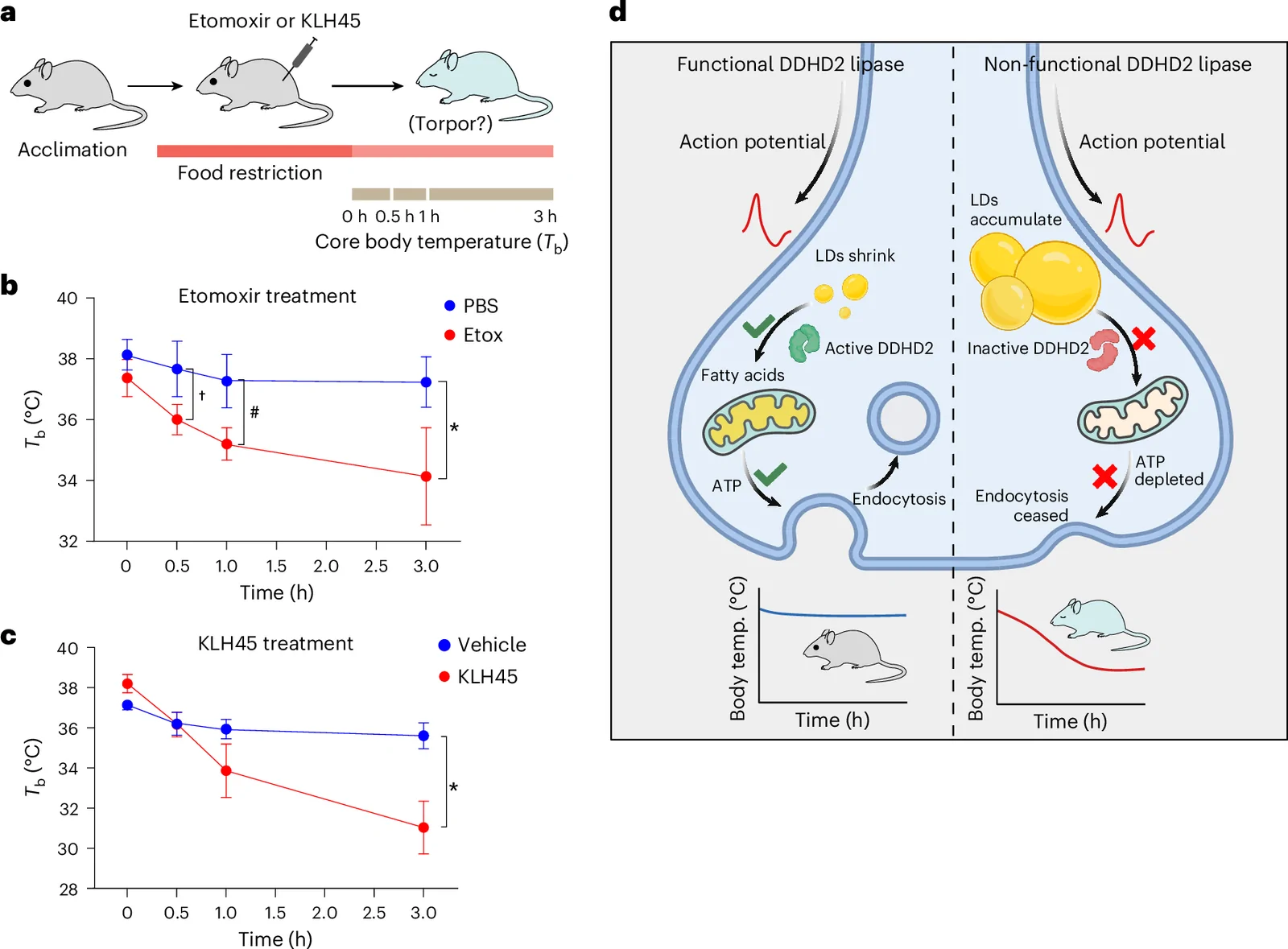
Inhibition of lipolysis in the brain induces torpor. **a**, Schematic representation of the experimental paradigm illustrating induction of torpor following intraperitoneal injection of etomoxir or KLH45. Environmentally acclimated (≥3 days) and individually housed mice were placed on food restriction for 3 h. The mice were injected with etomoxir or KLH45 and returned to their cages without food. Core body temperature (*T*_b_) was measured at regular intervals up to 3 h. **b**, *T*_b_ at 0, 0.5, 1 and 3 h after etomoxir injection. Mice injected with vehicle for etomoxir (PBS) were used as controls. Data are presented as means; error bars, s.e.m. *P* values (^†^*P* = 0.027; ^#^*P* = 0.0129; *P = 0.0216) were determined using an unpaired samples two-tailed *t*-test for *n* = 3 in both control (PBS) and etomoxir conditions. **c**, *T*_b_ at 0, 0.5, 1 and 3 h after KLH45 injection. Mice injected with vehicle for KLH45 (18:1:1 saline/ethanol/polyethylene glycol monooleyl ether) were used as controls. Data are presented as means; error bars, s.e.m. *P* value (*P = 0.0187) was determined using an unpaired samples two-tailed *t*-test for *n* = 3 in both control (vehicle) and KLH45 conditions. Mice injected with etomoxir or KLH45 (red) exhibited a significant drop in their core body temperature compared to controls (blue). **d**, Schematic summary of the mechanism of DDHD2-dependent FA fuelling of synaptic function to prevent a torpor-like state. Asterisk (*), hash (#) and dagger (†) symbols are used to distinguish between different groups of statistically significant comparison. Created in BioRender.com. Source data

Torpor is an adaptive hypothermic state in warm-blooded animals that can be triggered by a combination of metabolic stresses, including food restriction and cold environments. It is initiated by the activity of a subset of hypothalamic neurons in the brain^26^. We reasoned that if neurons are constantly making use of a flux of FAs for β-oxidation, blocking this pathway would also constitute an energy deficit that might drive torpor induction. To test this idea, 8–10-week-old male mice were injected with either etomoxir (to block CPT1 activity and subsequent β-oxidation) or a vehicle control, and the mice were food-restricted (Fig. 5a). Remarkably, following etomoxir injection, the core body temperature of the mice dropped rapidly (~3 °C within the first hour) (Fig. 5b). As CPT1 is widely expressed in all tissues, to determine whether this was a neuron-specific acute dependence on FA fuelling, we carried out the same protocol with KLH45. Given that in DDHD2 knockout mice, the only tissue to show even a small accumulation of TGs outside of the brain is adipose tissue, and that we show that KLH45 leads to no measurable accumulation of LDs in astrocytes, we reasoned that any impact of KLH45 in vivo would probably be attributable to its impact on neuron function. These experiments showed that as with CPT1 blockade, acute blockade of neuronal TG lipase activity rapidly led mice into a torpor state, as reflected by an almost 7 °C drop in core body temperature over a 3 h period (Fig. 5c). Together, these data indicate that at least certain neuron types in the brain are partly reliant on LDs as a constant source of ongoing β-oxidation, which helps prevent the onset of a torpor-like state (Fig. 5d).

## Discussion

The discovery that loss of DDHD2 function leads to a profound accumulation of LDs in neurons throughout the brain overturns a long-held view that neurons do not store, and therefore do not use, TGs as part of their metabolic programme. Given that loss of DDHD2 function in humans and mice leads to significant cognitive impairment, these results imply that this branch of metabolic support serves a critical role in sustaining neuron health and function, but previous studies did not examine what function these LDs, normally consumed by DDHD2, have. Here, we demonstrate conclusively that although LD abundance in neurons is very low at steady state, in agreement with most previous literature observations, this is the result of a constant flux of FAs through the LD pathway. Remarkably, we find that this pathway is important for axons, as DDHD2 is present in nerve terminals and its inhibition leads to the appearance of mature LDs in a majority of nerve terminals. We show that this flux itself is controlled by neuronal electrical activity and that FAs, once liberated from TGs in LDs, are transferred into the mitochondrial matrix, which then supports mitochondria-based ATP production. Both humans^8^ and mice^6^ harbouring genetic lesions in DDHD2 show a large accumulation of lipids in the brain and suffer from a spectrum of neurological disorders. Although it is possible that the presence of LDs themselves lead to an impairment in neuronal function that might underlie these disorders, our in vivo experiments indicated that loss of the flux of FAs from TGs leads to very rapid onset of changes in physiological state, more consistent with bioenergetic role for the FAs than a negative role of LDs.

Our work now brings into focus several key questions that are central to brain bioenergetics; in particular, the source of FAs that are normally used to build LDs and subsequently consumed by DDHD2 and the route for delivery of FAs into the neuron cytoplasm. One class of attractive candidates that might act as the extracellular conduit for FAs are lipoprotein particles, a common vehicle for shuttling sterols and TGs throughout the body. Astrocytes are well known to produce lipoprotein particles that are secreted into the extracellular space in the brain^27^. One of the main protein adaptors on astrocyte-derived lipoprotein particles is ApoE, the allelic variation of which is known to significantly impact Alzheimer’s disease risk^28^. Efficient extraction of TGs from lipoprotein particles generally requires that the receiving cell express lipoprotein lipase, a secreted protein that normally acts at the surface of a cell to extract FAs for delivery to surface FA transporters. Notably, most neurons in the brain express lipoprotein lipase, and genetic deletion of neuronal LPLs leads to a spectrum of neuronal dysfunction^29,30^. It has long been known that in muscle, FAs and glucose can co-regulate each other’s use as a fuel, such that one fuel is preferentially used and inhibits combustion of the other, known as the Randle Cycle^31,32^. It is intriguing to consider that such cross-regulation might be operational in neurons as well, and it will be interesting in the future to understand how this potential cross-regulation might in turn be impacted by electrical activity. In the future, it will be important to determine the cell biological routes of how TG-derived FAs are transferred to the surface of mitochondria and the underlying machineries that support this action, as well as to determine the suitability of lipoprotein particles as an extracellular route of TG delivery to neurons. It is also intriguing to consider that the ability of neurons to make use of LD-derived FAs for local presynaptic ATP production may become more crucial in ageing brains, as access to glucose in the brain might become more limited^33^.

## Methods

### Animals

Wild-type strains of Sprague Dawley rats (Charles River Laboratories, strain no. 400, RRID:RGD_734476) and C57BL/6J mice (Jackson Laboratory, strain no. 000664, RRID:IMSR_JAX:000664) were used to culture neurons and induce hypothermia, respectively. The animals were housed at an ambient temperature of 22–24 °C and relative humidity of 45–55% and were maintained on a 12 h light–dark cycle, with standard mouse chow and water provided ad libitum. All animal experiments were performed in accordance with the protocol reviewed and approved by the Institutional Animal Care and Use Committees of Weill Cornell Medicine, Rockefeller University, Yale University or UT Southwestern Medical Center.

### Chemicals

All chemicals used in the study were of analytical grade and procured from Sigma-Aldrich unless specified otherwise. The solutions and buffers were prepared with deionized water with a resistivity of 18.1 MΩ cm or above. The pharmacological inhibitors were dissolved in dimethylsulfoxide (Sigma-Aldrich, D2650) unless specified otherwise.

### Antibodies

The following antibodies were used: (1) for immunolabelling: DDHD2 (Proteintech, 25203-1-AP, RRID:AB_2879957), synapsin (Synaptic Systems, 106004, RRID:AB_1106784), TOMM20 (Sigma-Aldrich, WH0009804M1, RRID:AB_1843992), β-III tubulin (R&D Systems, MAB1195, RRID:AB_357520), NeuN (Abcam, ab104224, RRID:AB_10711040), GFAP (Abcam, ab4674, RRID:AB_304558), catalase (SanaCruz Biotech, sc-271803, RRID:AB_10708550), ERGIC3 (SanaCruz Biotech, sc-514611), GM130 (Cell Signaling Technology, 70767T); (2) for western blotting: CPT2 (Abcam, ab181114, RRID:AB_2687503) and GAPDH (Cell Signaling Technology, 5174S, RRID:AB_10622025).

### Primary cultured neurons and transfection

Glass coverslips were coated overnight with a 10 μg ml^−1^ solution of poly-ornithine (Sigma-Aldrich, P-3655). The hippocampal regions (CA1–CA3) were isolated from neonatal rat pups aged 0–1 day, without gender-based distinction. The dissociated hippocampal neurons were cultured in Minimum Essential Medium (ThermoFisher Scientific, 51200038) containing 29 mM glucose, 0.1 mg ml^−1^ bovine transferrin (Millipore, 616420), 24 μg ml^−1^ insulin (Millipore Sigma, I6634), 1% GlutaMAX (Thermo Fisher, 35050061), 5% fetal bovine serum (Atlanta Biologicals, S11510), 2% N-21 (R&D Systems, AR008) and 4 μM cytosine β-d-arabinofuranoside (Millipore Sigma, C6645). The cultures were maintained at 37 °C in a humidified incubator with 95% air and 5% CO_2_ for the next 21 days until being used for the experiments. The neurons were transfected using the calcium phosphate method after 7 days of plating. Imaging was performed between 7 and 14 days post transfection. Detailed stepwise protocols for hippocampal neuron culture and transfection are described elsewhere (https://doi.org/10.17504/protocols.io.ewov1qxr2gr2/v1).

### Immunohistochemistry with brain sections

Mice were anaesthetized with a ketamin–xylazine anaesthetic cocktail injection, perfused transcardially with 37 °C-warmed 4% formaldehyde + 0.125% glutaraldehyde in 0.1 M phosphate buffer, and the brains were kept in the same fixative overnight at 4 °C. Coronal vibratome-cut sections (25 μm thickness) were then blocked with a solution containing 2% normal goat serum, 2% BSA, 0.1 M phosphate buffer and 0.4% Triton X-100 for 1 h at room temperature (20–25 °C), incubated with primary antibodies (diluted in the same buffer) overnight at 4 °C, washed, incubated with Alexa-conjugated secondary antibodies for 2 h at room temperature and finally mounted with Prolong Gold antifade reagent with DAPI and sealed with nail polish. Images were acquired with the Yokogawa spinning disk field scanning confocal system with microlensing (×63, oil immersion objective lens, CSU-W1 SoRa, Nikon) controlled by NIS elements (Nikon) software (RRID:SCR_014329). A detailed stepwise protocol is described elsewhere (https://doi.org/10.17504/protocols.io.4r3l292kpv1y/v1).

### Immunocytochemistry with primary hippocampal neurons

Hippocampal neurons were sparsely cultured from post-natal day 0–1 pups on glass coverslips coated with poly-d-lysine (Sigma-Aldrich, P7886). When neurons reached 14 days in vitro, they were fixed using a solution of 4% PFA and 10% sucrose in PBS for 10 min at room temperature. The fixed neurons were rinsed three times with PBS (5 min each) and permeabilized with 0.25% Triton X-100 in PBS for 5 min, followed by three cycles of PBS washes (5 min each). The permeabilized neurons were blocked with 5% goat serum (Sigma-Aldrich, G9023) in PBS for 1 h at room temperature. The blocked neurons were gently washed once with PBS for 10 min. Primary antibodies were diluted (following the manufacturer’s instructions) in 5% goat serum prepared in 0.05% Triton X-100 containing PBS, applied to blocked neurons and incubated overnight at 4 °C in a humidified chamber. The following day, the neurons were washed three times with PBS (5 min each). Subsequently, secondary antibodies (conjugated to Alexa 488 or Alexa 546, Invitrogen) were applied to the neurons, typically in a 1:500 dilution prepared in a solution of 0.05% Triton X-100 in PBS, and incubated for 1 h at room temperature in a humidified chamber. Next, the neurons were subjected to two rounds of PBS wash, each for 5 min. To stain LDs, the third wash was performed for 10 min in PBS, containing 1 μg ml^−1^ BODIPY (Invitrogen, D3922) or 100 μM MDH (Abgent, SM1000a), followed by a gentle wash with PBS for 10 min. Finally, the glass coverslips containing stained neurons were mounted on glass slides using mounting media that contained BODIPY or MDH to compensate for any potential bleaching during laser microscopy. A detailed stepwise protocol is described elsewhere (https://doi.org/10.17504/protocols.io.eq2ly6j2egx9/v1).

### Electron microscopy with primary cultured neurons

The hippocampal neurons were dissociated and plated densely, confined within a 5 mm cylinder mounted on poly-ornithine-coated glass coverslips. The neurons were fixed for 1 h at room temperature using a solution containing 2.5% glutaraldehyde and 2 mM CaCl_2_ in 0.1 M sodium cacodylate buffer. The fixed neurons were washed four times, each for 5 min, with 0.1 M sodium cacodylate buffer containing 2 mM CaCl_2_. Subsequently, the neurons were post-fixed in 2% OsO_4_ and 1.5% K_4_Fe(CN)_6_ + 2 mM CaCl_2_ in 0.1 M sodium cacodylate buffer. The samples were en bloc stained with 2% aqueous uranyl acetate and 2 mM CaCl_2_ (J.T.Baker), dehydrated and embedded in Embed 812. Ultrathin sections of 50–60 nm thickness were observed in a Talos L 120 C transmission electron microscope at 80 kV. Final images were captured using Velox software (v.3.7.0.872-b0a7ba3a2f) and a 4K × 4K Ceta CMOS Camera (ThermoFisher Scientific). Unless otherwise specified, all reagents for electron microscopy were acquired from Electron Microscopy Sciences. A detailed stepwise protocol is described elsewhere (https://doi.org/10.17504/protocols.io.n92ldrmx8g5b/v1).

### Lipidomic analysis of isolated neuronal LDs

LDs were induced in the dissociated cortical neurons and purified using a sucrose density gradient. A detailed method for the induction, purification and biophysical analysis of the neuronal LDs is published elsewhere (https://doi.org/10.1101/2023.12.13.571527)^18^. The LD samples were subjected to MTBE extraction with spiked Avanti Splashmix as the internal standard. The organic phase was collected, dried and subsequently resuspended in 1-butanol. Then, 3 µl of this mixture was loaded onto an Agilent Poroshell 120 (EC-C18 2.7 µm, 1,000 bar, 2.1 × 100 mM) column using an Agilent 1290 Infinity II liquid chromatography system. Mobile phase A (60% acetonitrile, 40% H_2_O, 7.5 mM ammonium acetate) and mobile phase B (90% isopropanol, 10% acetonitrile, 7.5 mM ammonium acetate) were used to separate peaks for detection on an Agilent Quadrupole Time-Of-Flight 6546 mass spectrometer. The gradient started at 85% (15% B) and decreased to 70% A over 2 min and then to 52% A over 30 s. The gradient then slowly decreased to 18% A over 12.5 min and then 1% A over 1 min, and these concentrations were held for 4 min. The gradient was then restored to 85% A, and the column was washed for 5 min. Lipids were identified using MSDIAL (v.4.7) (PMID: 25938372, RRID:SCR_023076; https://systemsomicslab.github.io/compms/msdial/main.html). Each lipid species was quantified using the moles of corresponding class-specific lipid standards present in Splashmix.

### shRNA design and lentivirus production

shRNAs against the consensus coding sequence of target genes were designed using the available GPP Web Portal (https://portals.broadinstitute.org/gpp/public). The shRNA oligonucleotides, consisting of sense, hairpin loop and antisense, were flanked by restriction sites AgeI and EcoRI for subcloning. The following shRNAs were purchased from ThermoFisher: shRNA targeting DDHD2 (TRCN0000346842, ATAGTTTAGGTTCGCTTATAT) and shRNA targeting CPT2 (no. 1, GACCCAAAGTCTGAGTATAAT; no. 2, ACTAACTCAGCTGTATTTATT). The shRNA oligonucleotides were annealed and subcloned into the pLKO-TRC vector between the AgeI and EcoRI restriction sites. The resulting vector expressed shRNA under transcriptional control of the U6 promoter, along with a BFP (blue fluorescent protein) expressed under the control of the hPGK promoter to confirm the delivery of plasmids to cultured neurons. The pLKO plasmid (RRID:Addgene_191566) with desired oligonucleotides was delivered to HEK293FT (Thermo Fisher Scientific, R70007, RRID:CVCL_6911) cells along with two packaging plasmids, pPAX2 (Addgene, 12260, RRID:Addgene_12260) and pMD2 (Addgene, 12259, RRID:Addgene_12259), using jetPRIME DNA/siRNA transfection reagent (Polyplus, 101000027). The transfection media was replaced with virus production media 16 h after delivery of the plasmids. The supernatant containing lentivirus was collected at 46–48 h, filtered through a 0.45 μm syringe filter set and concentrated using Lenti-X concentrator (Takara Bio, 631231). The virus titre was measured using the Lenti-X GoStix Plus assay kit (Takara Bio, 631280). A detailed stepwise protocol is described elsewhere (https://doi.org/10.17504/protocols.io.dm6gp93ndvzp/v1).

### Construction of overexpression plasmids

vGlut1-pHluorin (Addgene, 207470, RRID:Addgene_207470) was used as previously published^22^. mCherry-TGNP-N-10 (Addgene, 55145, RRID:Addgene_55145) was procured from Addgene. mRuby–synapsin (Addgene, 187896, RRID:Addgene_187896) was cloned into pLV under the hSynapsin promoter using AgeI and SalI restriction sites. eGFP-DDHD2 was PCR-amplified from DDHD2_OHu23306C_pcDNA3.1(+)-N-eGFP (GenScript, Clone ID OHu23306) and cloned into the pLV under the hSynapsin promoter using XhoI and PmeI restriction sites. CPT2 was PCR-amplified from CPT2_OHu17939D_pcDNA3.1+/C-(k)DYK (GenScript, Clone ID OHu17939D) and cloned to the pLV under the hSynapsin promoter and carboxy-terminal Halo tag using XhoI and PmeI restriction sites. The Gibson assembly method was used with NEBuilder HiFi DNA Assembly Master Mix (New England Biolabs, E2621L) to assemble the desired fragments in the above plasmids. A detailed stepwise protocol is described elsewhere (https://doi.org/10.17504/protocols.io.36wgqd3y3vk5/v1).

### Western blot to confirm knockdown

Cortical neurons obtained from rat pups aged 0–1 days were cultured according to the above procedure on 35 mm dishes coated with 50 μg ml^−1^ poly-d-lysine. At 2 days in vitro, lentivirus was introduced to the cortical neurons. To extract proteins, neurons were scraped into RIPA lysis buffer (25 mM Tris-HCl pH 7.6, 150 mM NaCl, 1% NP-40, 1% sodium deoxycholate, 0.1% SDS) supplemented with complete protease inhibitor (Roche, 11697498001). The protein concentration in the lysed cytosol was quantified using the BCA method, following the manufacturer’s guidelines. The proteins in the samples were denatured using sample buffer containing SDS (BioRad, 1610747) and subsequently transferred to a PVDF membrane (Millipore Sigma, IPFL00010). These membranes were then blocked with a blocking buffer (LiCor, 927-60001) for 1 h at room temperature. After blocking, the membranes were subjected to overnight incubation with primary antibodies at 4 °C, followed by HRP-conjugated secondary antibodies (Invitrogen, 31430 and 31460) for 1 h at room temperature. Both the primary and secondary antibodies were diluted in LiCor blocking buffer according to the manufacturer’s recommendations. The HRP signal was detected using the ECL kit (ThermoFisher, 32109), and images were captured using the LiCor Odyssey Fc system. A detailed stepwise protocol is described elsewhere (https://doi.org/10.17504/protocols.io.3byl4wqdjvo5/v1).

### FA induction to hippocampal neurons

Long-chain free FAs, owing to their low solubility in aqueous medium, were complexed with albumin to facilitate their delivery to cells. FA-free BSA (Sigma-Aldrich, A8806) was overlayed on 0.1 M Tris (pH 8) at 37 °C and allowed to dissolve completely. Oleic acid (Sigma-Aldrich, O1383) was added to the Tris-BSA solution in a 6:1 molar ratio and stirred gently at 37 °C to prepare the oleate–BSA complex. This complex was subsequently passed through a 0.22 µm syringe filter before being used in cell culture. Palmitic acid, in contrast to oleic acid, is solid at room temperature. FA-free BSA was dissolved in 0.9% NaCl at 40 °C, and palmitic acid (Sigma-Aldrich, P0500) was solubilized in 0.1 M NaOH by heating to 70 °C. These two solutions were then combined in a 5:1 molar ratio (palmitate–BSA) and vortexed at 40 °C until the solution became turbid. Similar to the oleate complex, the palmitate complex was also passed through a 0.22 µm syringe filter before being used in cell culture. The oleate complex was used within 3 months after preparation, whereas the palmitate complex was freshly prepared and maintained at 37 °C during the experiment. A detailed stepwise protocol is described elsewhere (https://doi.org/10.17504/protocols.io.n2bvj93jxlk5/v1).

### BODIPY 558/568 C12 tracing in neurons

Sparsely cultured hippocampal neurons were pulsed with 10 μM BODIPY 558/568 C12 (Cayman Chemical, 27014; referred in this paper as Red-C12) for 24 h in 5 μM KLH45 (Sigma-Aldrich, SML1998) containing feeding media. The neurons were transferred to complete feeding media without Red-C12 for 1 h to incorporate the Red-C12 into neuronal LDs. The neurons were subsequently washed and transferred to HBSS and chased for the defined period (0–4.5 h) either in the absence or presence of 10 μM etomoxir or 5 μM TTX. Then, 30 min before the end of incubation time, 50 nM MitoTracker Green FM (Thermo Scientific, M7514) was added to the media and the neurons were returned to the incubator. The neurons were washed once with HBSS and transferred to fresh HBSS (with inhibitors) and conditioned in an incubator. The coverslips with the neurons were mounted on a custom-designed chamber for imaging on a confocal microscope. A detailed stepwise protocol is described elsewhere (https://doi.org/10.17504/protocols.io.dm6gp9328vzp/v1).

### Imaging with live neurons

All epifluorescence time-lapse images were acquired using a custom-modified Zeiss Axiovert 200 microscope equipped with laser illumination, an Andor iXon camera (model DU-897U-CS0-BVF) and a ×40, 1.3 NA Fluar Zeiss objective. To control the OBIS solid-state 488 nm and 561 nm lasers, acousto-optic tunable filters were used on the light path. To control the buffer flow to the neurons, coverslips with cultured and transfected neurons were mounted with a laminar flow perfusion system maintained at 37 °C. The perfusion solution consisted of Tyrode’s solution, comprising 119 mM NaCl, 2.5 mM KCl, 2 mM CaCl_2_, 2 mM MgCl_2_, 50 mM HEPES (pH 7.4) and 0–5 mM glucose. It was supplemented with 10 mM 6-cyano-7-nitroquinoxalibe-2, 3-dione (CNQX) and 50 mM d,l-2-amino-5-phosphonovaleric acid (acquired from Sigma-Aldrich) to suppress post-synaptic responses. For experiments involving FAs, the feeding media were supplemented with 150 μM BSA-complexed palmitic acid for 4 h. The Tyrode’s solution containing 100 μM BSA-complexed palmitic acid was perfused to the neurons mounted on a temperature-controlled objective at 37 °C. The following concentrations of the pharmacological inhibitors were added to the appropriate Tyrode’s solution and perfused for 5–10 min before electrical stimulations: KLH45 (Sigma-Aldrich, SML1998), 5 µM; oligomycin (Sigma-Aldrich, O4876), 2 µM; etomoxir (Sigma-Aldrich, E1905), 20 µM; TTX (Abcam, ab120055), 2 µM; and koningic acid (also known as heptelidic acid; Cayman, 14079), 10 μM. Action potentials in neurons were triggered by brief 1 ms pulses, generating a field potential of ~10 V cm^−1^, using platinum–iridium electrodes. Image sequences acquired on an epifluorescence microscope were analysed using ImageJ Fiji software (v.1.54 f, RRID:SCR_003070; https://imagej.net) embedded with plugin time series analyzer (v.3, RRID:SCR_014269; https://imagej.nih.gov/ij/plugins/time-series.html). Approximately 20–25 regions of interest, each covering an area of 12–18 μm^2^ and corresponding to responsive synaptic boutons, were manually selected for fluorescence measurement over time.

Control untreated and KLH45-induced neurons were incubated with 5 μM CellROX (Fisher Scientific, C10444) for 30 min to assess cellular reactive oxygen or oxidative stress. Parallelly, control neurons were treated with 2 μM Rotenone (Sigma-Aldrich, R8875) for 3 h to induce reactive oxygen species before staining with CellROX. Neurons were incubated with 100 nM LysoTracker-Red (Fisher Scientific, L7528) for 30 min to visualize lysosomes in the soma before immunostaining with DDHD2 antibody. Mitochondria were stained with 50 nM MitoTracker Green (ThermoScientific, M7514) and JF585 dye for 30 min. CellROX, LysoTracker-Red and MitoTracker Green were washed with the growth media before mounting on coverslips. The images were acquired using a Zeiss LSM 880 32-channel Airyscan confocal microscope or a Zeiss Axiovert 200 microscope under appropriate excitation and emission settings. A detailed stepwise protocol is described elsewhere (https://doi.org/10.17504/protocols.io.n92ldrmqog5b/v1).

### Hypothermia induction in mice

Hypothermia was induced with 30 mg kg^−1^ etomoxir or 40 mg kg^−1^ KLH45 by intraperitoneal injection in 8–10-week-old wild-type C57BL/6 male mice. Etomoxir was dissolved in PBS. KLH45 was dissolved in emulphor composed of 18:1:1 saline:ethanol:polyethylene glycol monooleyl ether (Fisher Scientific, P071325G). Respective vehicles were used as controls. Body temperature was measured 0, 0.5, 1 and 3 h after intraperitoneal injection using a rectal probe for mice (Kent Scientific, RET-3). Mice were placed on food restriction 3 h before injection and were returned to their cages without feed after injection. A detailed stepwise protocol is described elsewhere (https://doi.org/10.17504/protocols.io.6qpvr937bvmk/v1).

### Statistical analysis

All synaptic function experiments were conducted on primary neurons prepared from neonatal animals without sex bias. All experiments were performed in at least three independent biological replicates; the exact number of replicates is specified in the corresponding figure legends. No formal statistical method was used to predetermine sample size, but our sample sizes were similar to previously published peer-reviewed studies. Neurons in functional tests and microscopy were selected from random field of views to avoid any artefacts and improve the robustness of the data. The investigators were not blinded to the experimental conditions during data collection and analysis. No data were excluded from analysis, except for the neurons that appeared non-responsive in control conditions during synaptic function assays. Unless otherwise indicated, the data points were assumed to be normally distributed, but this was not formally tested. The datasets (traces and data points) are presented as means ± s.e.m. unless indicated otherwise in the figure legends. Male mice aged 8–10 weeks were used for the torpor experiments. Statistical analyses were performed using Origin software (v.9.800200, v.9.900225 and v.10.00154; RRID:SCR_014212; https://www.originlab.com/index.aspx?go=PRODUCTS/Origin) or GraphPad Prism (v.6.0; RRID:SCR_002798; http://www.graphpad.com). For group comparisons, statistical analyses were conducted using an unpaired samples two-tailed *t*-test or one-way ANOVA, followed by Tukey’s multiple comparison test. Statistical significance (*P* values) is denoted as follows: ns, *P* > 0.05; *P ≤ 0.05; **P ≤ 0.01; ***P ≤ 0.001; ****P ≤ 0.0001. Significance values are also indicated as appropriate in the figure legends.

### Reporting summary

Further information on research design is available in the Nature Portfolio Reporting Summary linked to this article.

## Supplementary information

Reporting Summary

## Source data

Source Data Fig. 1 Statistical source data.

Source Data Fig. 2 Statistical source data.

Source Data Fig. 3 Statistical source data.

Source Data Fig. 4 Statistical source data.

Source Data Fig. 5 Statistical source data.

Source Data Extended Data Fig./Table 1 Statistical source data.

Source Data Extended Data Fig./Table 2 Statistical source data.

Source Data Extended Data Fig./Table 3 Statistical source data.

Source Data Extended Data Fig./Table 4 Statistical source data.

Source Data Extended Data Fig./Table 5 Statistical source data.

Source Data Extended Data Fig./Table 6 Statistical source data.

Source Data Extended Data Fig./Table 7 Statistical source data.

Source Data Extended Data Fig./Table 8 Statistical source data.

Source Data Extended Data Fig./Table 9 Statistical source data.

Source Data Extended Data Fig./Table 10 Statistical source data.

Source Data Extended Data Fig./Table 10 Unprocessed western blots.

## Data Availability

Data supporting the study are available as source data files. Raw and unprocessed datasets generated for the study have been deposited in Zenodo and are publicly accessible at https://doi.org/10.5281/zenodo.15176704 (ref. ^34^) and https://doi.org/10.5281/zenodo.15115578 (ref. ^35^). Source data are provided with this paper.
