# Supplementary material for: Triglycerides are an important fuel reserve for synapse function in the brain
Source: Nat Metab. 2025 Jul 1;7(7):1392–403. doi: 10.1038/s42255-025-01321-x (PMC12286841; doi:10.1038/s42255-025-01321-x)
Supplement: Supplementary file 1 — Reporting Summary [file 42255_2025_1321_MOESM1_ESM.pdf]

nature portfolio

Corresponding author(s): Timothy A. Ryan

Last updated by author(s): Apr 23, 2025

Reporting Summary

Nature Portfolio wishes to improve the reproducibility of the work that we publish. This form provides structure for consistency and transparency in reporting. For further information on Nature Portfolio policies, see our Editorial Policies and the Editorial Policy Checklist.

Statistics

For all statistical analyses, confirm that the following items are present in the figure legend, table legend, main text, or Methods section.

n/a	Confirmed	
☐	☒	The exact sample size (*n*) for each experimental group/condition, given as a discrete number and unit of measurement
☐	☒	A statement on whether measurements were taken from distinct samples or whether the same sample was measured repeatedly
☐	☒	The statistical test(s) used AND whether they are one- or two-sided<br>*Only common tests should be described solely by name; describe more complex techniques in the Methods section.*
☐	☒	A description of all covariates tested
☐	☒	A description of any assumptions or corrections, such as tests of normality and adjustment for multiple comparisons
☐	☒	A full description of the statistical parameters including central tendency (e.g. means) or other basic estimates (e.g. regression coefficient) AND variation (e.g. standard deviation) or associated estimates of uncertainty (e.g. confidence intervals)
☐	☒	For null hypothesis testing, the test statistic (e.g. *F*, *t*, *r*) with confidence intervals, effect sizes, degrees of freedom and *P* value noted<br>*Give P values as exact values whenever suitable.*
☒	☐	For Bayesian analysis, information on the choice of priors and Markov chain Monte Carlo settings
☒	☐	For hierarchical and complex designs, identification of the appropriate level for tests and full reporting of outcomes
☐	☒	Estimates of effect sizes (e.g. Cohen's *d*, Pearson's *r*), indicating how they were calculated

Our web collection on statistics for biologists contains articles on many of the points above.

Software and code

Policy information about availability of computer code

Data collection	ANDOR Solis (v4.32.30000.0) and Zen (v3.10) softwares were used to acquire fluorescent images. EM images were captured using Velox software (v3.7.0.872-b0a7ba3a2f). Western blots were imaged on Odyssey Fc imaging system (LI-COR, Serial-1328) using Image Studio (v5.2).
Data analysis	All western blots and fluorescent images were processed and analyzed with imagej/Fiji (v1.54f) software. Origin software (2021 v9.800200, 2022 v9.900225 & 2023 v10.00154), and GraphPad Prism (v6.0) were used to plot graphs and statistical analyses.

For manuscripts utilizing custom algorithms or software that are central to the research but not yet described in published literature, software must be made available to editors and reviewers. We strongly encourage code deposition in a community repository (e.g. GitHub). See the Nature Portfolio guidelines for submitting code & software for further information.

Data

Policy information about availability of data

All manuscripts must include a data availability statement. This statement should provide the following information, where applicable:

- Accession codes, unique identifiers, or web links for publicly available datasets
- A description of any restrictions on data availability
- For clinical datasets or third party data, please ensure that the statement adheres to our policy

Source data files associated with the Main and Extended Data Figures are being provided with this manuscript. Raw data files and images associated with the Main and Extended Data Figures are publicly available on Zenodo (DOI:10.5281/zenodo.15115578 & DOI:10.5281/zenodo.15176704) via ASAP Open Science Policy.

Research involving human participants, their data, or biological material

Policy information about studies with human participants or human data. See also policy information about sex, gender (identity/presentation), and sexual orientation and race, ethnicity and racism.

Reporting on sex and gender	N/A
Reporting on race, ethnicity, or other socially relevant groupings	N/A
Population characteristics	N/A
Recruitment	N/A
Ethics oversight	N/A

Note that full information on the approval of the study protocol must also be provided in the manuscript.

Field-specific reporting

Please select the one below that is the best fit for your research. If you are not sure, read the appropriate sections before making your selection.

☒ Life sciences ☐ Behavioural & social sciences ☐ Ecological, evolutionary & environmental sciences

For a reference copy of the document with all sections, see nature.com/documents/nr-reporting-summary-flat.pdf

Life sciences study design

All studies must disclose on these points even when the disclosure is negative.

Sample size	No statistical method was use to predetermine the sample size. Our sample sizes were similar to those used in previously published, peer-reviewed studies relevant to this work (PMID: 39167658, PMID: 38748581). The sample size and replications for each experiment is specified in the figure legends.
Data exclusions	No data were excluded from analysis, except for the neurons that appeared non-responsive in control conditions during synaptic function assays.
Replication	All experiments were reproducible. The neonatal animals used in the experiments were from multiple litters sufficient to demonstrate statistical significance. The number of replicates used in the experiments are mentioned in the corresponding figure legends and statistical analysis section of method.
Randomization	Animals were randomly assigned to control or different experimental groups without any bias.
Blinding	Data analysis was not performed blind to the experimental conditions. All cell-based experiments were performed under same conditions, except for the indicated experimental variations, and analysis was performed ensuring appropriate internal controls. Additionally, quantification of the data relied upon software-based automatic calculations.

Reporting for specific materials, systems and methods

We require information from authors about some types of materials, experimental systems and methods used in many studies. Here, indicate whether each material, system or method listed is relevant to your study. If you are not sure if a list item applies to your research, read the appropriate section before selecting a response.

Materials & experimental systems

n/a	Involved in the study	
☐	☒	Antibodies
☐	☒	Eukaryotic cell lines
☒	☐	Palaeontology and archaeology
☐	☒	Animals and other organisms
☒	☐	Clinical data
☒	☐	Dual use research of concern
☒	☐	Plants

Methods

n/a	Involved in the study	
☒	☐	ChIP-seq
☒	☐	Flow cytometry
☒	☐	MRI-based neuroimaging

Antibodies

Antibodies used

Primary antibodies:
DDHD2 (Proteintech, 25203-1-AP, RRID:AB_2879957, For IF 1:100, IHC 1:200),
Synapsin (Synaptic Systems, 106004; RRID:AB_1106784, For IF 1:500, IHC 1:250),
TOMM20 [4F3](Sigma Aldrich, WH0009804M1; RRID:AB_1843992, For IF 1:500),
β-III tubulin [Tuj-1] (R & D systems, MAB1195; RRID:AB_357520, For IF 1:1000),
NeuN [1B7] (Abcam, ab104224; RRID:AB_10711040, For IF 1:500),
GFAP (Abcam, ab4674; RRID:AB_304558, For IF 500),
Catalase [H9] (Santa Cruz Biotech, sc-271803; RRID:AB_10708550, For IF 1:200),
ERGIC3 [E3] (Santa Cruz Biotech, sc-514611, For IF 1:200),
GM130 [E9Z6S] (Cell Signaling tech, 70767, For IF 1:200),
CPT2 [EPR13626] (Abcam, ab181114; RRID:AB_2687503, For WB 1:2000),
GAPDH [D16H11] (Cell Signaling Technology, 5174; RRID:AB_10622025, For WB 1:2000),

Secondary Antibodies:
Alexa Fluor 488 Goat Anti-Mouse IgG (H+L) (Thermo Fisher Scientific, A-11029; RRID:AB_2534088, For IF 1:500)
Alexa Fluor 488 Goat Anti-Rabbit IgG (H+) (Thermo Fisher Scientific, A-11034; RRID:AB_2576217, For IF 1:500)
Alexa Fluor 546 Goat Anti-Guinea Pig IgG (H+L) (Thermo Fisher Scientific, A-11074; RRID:AB_2534118, For IF 1:500)
Alexa Fluor 555 Goat anti-Chicken IgY (H+L) (Thermo Fisher Scientific, A-21437, RRID:AB_2535858, For IF 1:500)
Alexa Fluor 647 Goat anti-Rabbit IgG (H+L) (Thermo Fisher Scientific, A-21245; RRID:AB_2535813, For IF 1:500)
Goat anti-Rabbit IgG (H+L) Poly-HRP Secondary Antibody, HRP (Thermo Fisher Scientific, 32260; RRID:AB_1965959, For WB 1:5000)

Validation

All the antibodies used in this study were commercially procured from the listed sources. The antibodies were either validated by supplier (as described and reported in the product information), previous studies, or present study. The validations were based on over-expression or knockdown followed by IF or western blotting of the respective protein bands of specific molecular weight.

- DDHD2 [Proteintech, 25203-1-AP, RRID:AB_2879957, Previously validated by knockdown and western blotting (PMID: 31786011, PMID: 38316990). In this study, we further validated the antibody by shRNA-based knockdown followed by IF],
- Synapsin [Synaptic Systems, 106004; RRID:AB_1106784, Vendor: https://sysy.com/products/wb/image-thumb__375__application-full/106004-wb.pjpeg],
- TOMM20 [4F3] [Sigma Aldrich, WH0009804M1; RRID:AB_1843992, Vendor: https://www.sigmaaldrich.com/deepweb/assets/sigmaaldrich/product/images/198/270/7dd525d5-b629-4be4-b39b-f260916185e8/800/7dd525d5-b629-4be4-b39b-f260916185e8.jpg],
- β-III tubulin [Tuj-1] [R & D systems, MAB1195; RRID:AB_357520, Resource Watch (ID: 352599), https://scicrunch.org/ResourceWatch/Search?q=AB_357520, Vendor: https://www.bio-techne.com/p/antibodies/neuron-specific-beta-iii-tubulin-antibody-tuj-1_mab1195?utm_source=antibodyregistry&utm_medium=referral&utm_campaign=product&utm_term=primaryantibodies#tab-citations_reviews]
- NeuN [1B7] [Abcam, ab104224; RRID:AB_10711040, Resource Watch (ID: 349920, 40), https://scicrunch.org/ResourceWatch/Search?q=AB_10711040, Vendor: https://www.abcam.com/en-us/products/primary-antibodies/neun-antibody-1b7-neuronal-marker-ab104224#]
- GFAP [Abcam, ab4674; RRID:AB_304558, Vandor: https://www.abcam.com/en-us/products/primary-antibodies/gfap-antibody-ab4674#],
- Catalase [H9] [Santa Cruz Biotech, sc-271803; RRID:AB_10708550, PMCID: PMC7243027, Vendor: https://www.scbt.com/p/catalase-antibody-h-9#citations],
- ERGIC3 [E3] [Santa Cruz Biotech, sc-514611, Vendor: https://media.scbt.com/product/ergic-3-antibody-e-3-western-blotting_32_47_z_324799.jpg],
- GM130 [E9Z6S] [Cellsignalling tech, 70767, PMCID: PMC11951976, DOI: 10.34133/research.0644],
- CPT2 [EPR13626] [Abcam, ab181114; RRID:AB_2687503, PMCID: PMC10447235, DOI: 10.1038/s42255-023-00835-6, Vendor: https://www.abcam.com/en-us/products/primary-antibodies/cpt2-antibody-epr13626-c-terminal-ab181114#overlay=images; In this study, we further validated the antibody by shRNA-based knockdown followed by WB],
- GAPDH [D16H11] (Cell Signaling Technology, 5174; RRID:AB_10622025, Vendor: https://www.cellsignal.com/products/primary-antibodies/gapdh-d16h11-xp-rabbit-mab/5174?index=1&application=all),

Eukaryotic cell lines

Policy information about cell lines and Sex and Gender in Research

Cell line source(s)

HEK-293FT (Thermo Fisher Scientific, R70007, RRID:CVCL_6911, Vendor: https://www.thermofisher.com/order/catalog/product/R70007), Primary hippocampal and cortical neurons were derived from neonatal rodents of both sexes.

Authentication

Cell line obtained were not authenticated.

Mycoplasma contamination

Cell line was checked for mycoplasma contamination using PCR-based kit (Millipore Sigma, MP0035-1KT) and confirmed negative before use.

Commonly misidentified lines
(See ICLAC register)

No commonly misidentified cell lines were used in this study.

Animals and other research organisms

Policy information about studies involving animals; ARRIVE guidelines recommended for reporting animal research, and Sex and Gender in Research

Laboratory animals	Wild-type strains of Sprague Dawley rats (Strain code: 400, RRID: RGD_734476) and C57BL/6J mice (Strain code: 000664, RRID: MSR_JAX:000664) were sourced from Charles River Laboratories and Jackson Laboratory, respectively. Animals were housed under pathogen-free condition, at an ambient temperature of 20-24°C, relative humidity of 45-55%, and maintained on a 12 hour light and dark cycle. Standard rodent chow and water were provided ad libitum.<br>Neonatal (P0-1) Sprague Dawley rat C57BL/6J mice pups were used to culture primary hippocampal and cortical neurons. Adult male C57BL/6J mice, aged 8-10 weeks, were used for Torpor induction experiments.
Wild animals	No wild animals were used in this study.
Reporting on sex	Gender was not considered for primary culture of neurons. Male mice were used for torpor experiments.
Field-collected samples	No field-collected samples were used in this study.
Ethics oversight	All animal experiments were performed in accordance with the protocol reviewed and approved by the Institutional Animal Care and Use Committee (IACUC) of Weill Cornell Medicine, Rockefeller University, Yale University and UT Southwestern Medical Center.

Note that full information on the approval of the study protocol must also be provided in the manuscript.

Plants

Seed stocks	N/A
Novel plant genotypes	N/A
Authentication	N/A
