# Supplementary material for: Triglycerides are an important fuel reserve for synapse function in the brain
Source: Nat Metab. 2025 Jul 1;7(7):1392–403. doi: 10.1038/s42255-025-01321-x (PMC12286841; doi:10.1038/s42255-025-01321-x)

Extended Data Fig. 10b

Western Blot of CPT2 (Abcam, Cat# ab181114) with ladder (ThermoFisher, Cat# 26619) overlayed

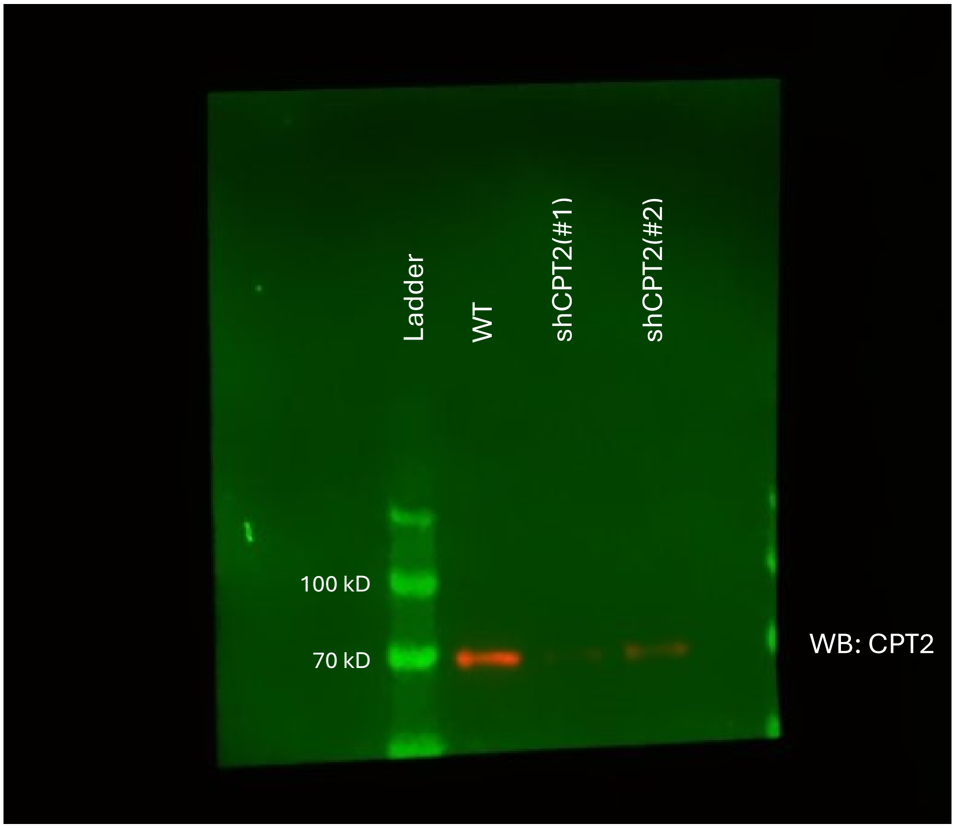

Western Blot for GAPDH (Cell Signaling Technology, Cat# 5174S) with ladder (ThermoFisher, Cat# 26619) overlayed

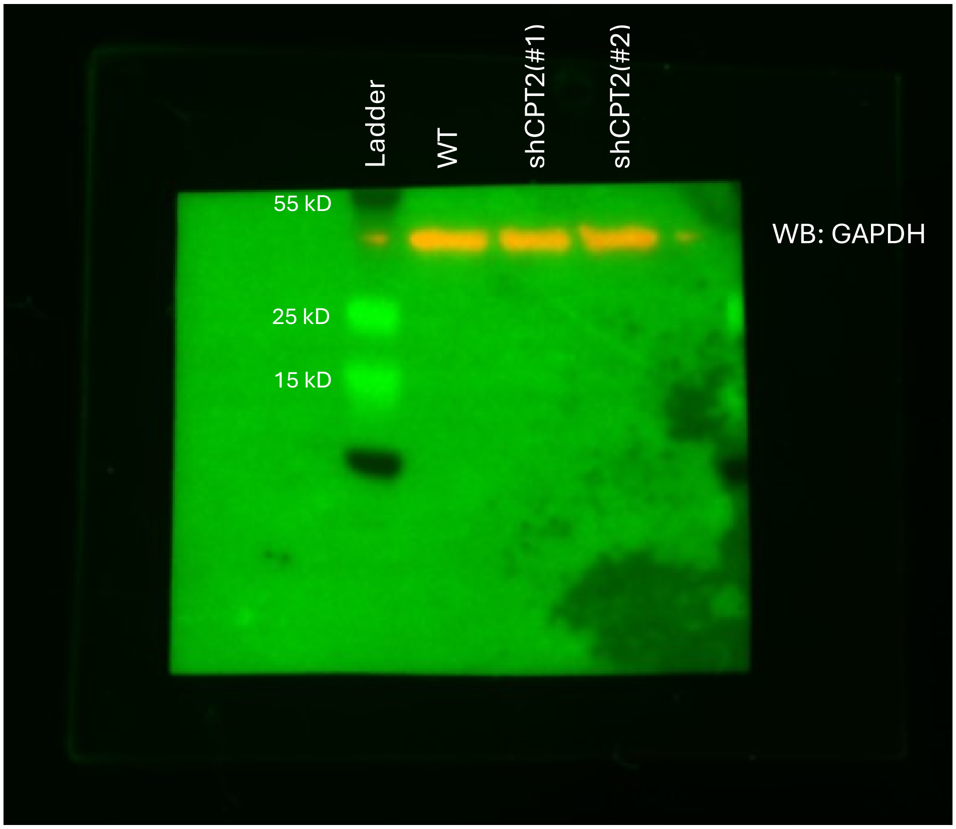

Supplement: Supplementary file 17 — Unprocessed western blots. [file 42255_2025_1321_MOESM17_ESM.pdf]
